# Chain-like gold nanoparticle clusters for multimodal photoacoustic microscopy and optical coherence tomography enhanced molecular imaging

**DOI:** 10.1038/s41467-020-20276-z

**Published:** 2021-01-04

**Authors:** Van Phuc Nguyen, Wei Qian, Yanxiu Li, Bing Liu, Michael Aaberg, Jessica Henry, Wei Zhang, Xueding Wang, Yannis M. Paulus

**Affiliations:** 1 Department of Ophthalmology and Visual Sciences, University of Michigan, Ann Arbor, MI 48105 USA; 2 IMRA America, Inc., 1044 Woodridge Ave., Ann Arbor, MI 48105 USA; 3 Department of Biomedical Engineering, University of Michigan, Ann Arbor, MI 48105 USA; 4 NTT-Hitech Institutes, Nguyen Tat Thanh University, Ho Chi Minh City, Vietnam

**Keywords:** Retinal diseases, Experimental models of disease, Biomedical engineering, Nanoparticles

## Abstract

Colloidal gold nanoparticles (GNPs) serve as promising contrast agents in photoacoustic (PA) imaging, yet their utility is limited due to their absorption peak in the visible window overlapping with that of hemoglobin. To overcome such limitation, this report describes an ultrapure chain-like gold nanoparticle (CGNP) clusters with a redshift peak wavelength at 650 nm. The synthesized CGNP show an excellent biocompatibility and photostability. These nanoparticles are conjugated with arginine-glycine-aspartic acid (RGD) peptides (CGNP clusters-RGD) and validated in 12 living rabbits to perform multimodal photoacoustic microscopy (PAM) and optical coherence tomography (OCT) for visualization of newly developed blood vessels in the sub-retinal pigment epithelium (RPE) space of the retina, named choroidal neovascularization (CNV). The PAM system can achieve a 3D PAM image via a raster scan of 256 × 256 pixels within a time duration of 65 s. Intravenous injection of CGNP clusters-RGD bound to CNV and resulted in up to a 17-fold increase in PAM signal and 176% increase in OCT signal. Histology indicates that CGNP clusters could disassemble, which may facilitate its clearance from the body.

## Abstract (web summary)

This manuscript presents ultrapure chain-like gold nanoparticle clusters with red shifted absorption and shows their potential for in vivo imaging in living rabbits. The nanoparticles demonstrate a 17-fold increase in photoacoustic microscopy signal and 176% increase in optical coherence tomography signal.

## Introduction

Gold nanoparticles (GNPs) serve as promising contrast agents for photoacoustic (PA) vascular and tumor imaging with a high signal. However, their clinical utility is limited due to the overlapping of their peak wavelength of the localized surface plasmon resonance (LSPR) with that of hemoglobin. As biological tissues have a lower absorption rate for light in the near-infrared (NIR) range, a contrast agent which absorbs NIR radiation would allow for selective visualization of targeted structures and enhanced visualization of the contrast agent with the least background noise^1^. GNPs of different shapes, such as sphere (>100 nm), rod, star, shell, and thin plate have been widely investigated as NIR contrast agents for photoacoustic and optical coherence tomography (OCT) imaging^2–7^. The use of these GNPs has been shown to improve molecular imaging. Unfortunately, these GNPs are often synthesized with the use of cetyltrimethylammonium bromide (CTAB) and are large in size, leading to long-term toxicity^8^. For in vivo PA imaging applications, a critical size limit for GNPs is about 100 nm^1,9^. Additionally, these nanoparticles traditionally have a poor thermal stability, unsatisfactory tissue delivery rate, insufficient delivery efficiency, short circulation time, and complexity in leaking from vessels^1,10^.

Therefore, the developed contrast agents should be smaller than 100 nm, non-toxic, strong absorbers, exhibit great scattering at NIR wavelengths, photostable, biodegradable, and capable of evading clearance via the human reticuloendothelial system (RES) to prolong circulation time^1^. Research for achieving these goals has already commenced. Several investigations have described surface-modifications of GNPs with various substances such as thiolate chitosan and polyelectrolyte to reduce potential cytotoxic effects^11,12^. Furthermore, to shift LSPR wavelengths of GNPs from the visible range to the NIR range while maintaining a small nanoparticle size, GNPs have been conjugated with Prussian blue or coated with silica^9,11^. Yet, these GNPs have been shown to easily aggregate in physiological saline and demonstrate poor thermal stability, resulting in a low photoacoustic signal and low signal to noise ratio. Herein, we present ultrapure, chain-like GNP (CGNP) clusters, fabricated through assembling spherical GNPs produced physically using femtosecond pulsed laser ablation of bulk gold target in deionized (DI) water to avoid toxicity associated with precursors used in chemical synthesis. Following the fabrication of CGNP clusters they were conjugated with linear arginine–glycine–aspartate (RGD) peptides (CGNP clusters-RGD) for molecular imaging. To validate our CGNP clusters-RGD as PA molecular imaging agents and to demonstrate its safety and biocompatibility, we performed in vivo PA imaging using two rabbit models of choroidal neovascularization (CNV): (1) retinal vein occlusion (RVO) induced via photocoagulation, and (2) subretinal injection of vascular endothelial growth factor (VEGF-165). In all these in vivo studies, CGNP clusters-RGD were intravenously injected via the rabbits’ marginal ear veins.

## Results

### Characterization and in vitro optical properties of CGNPs

**Fig. 1:**
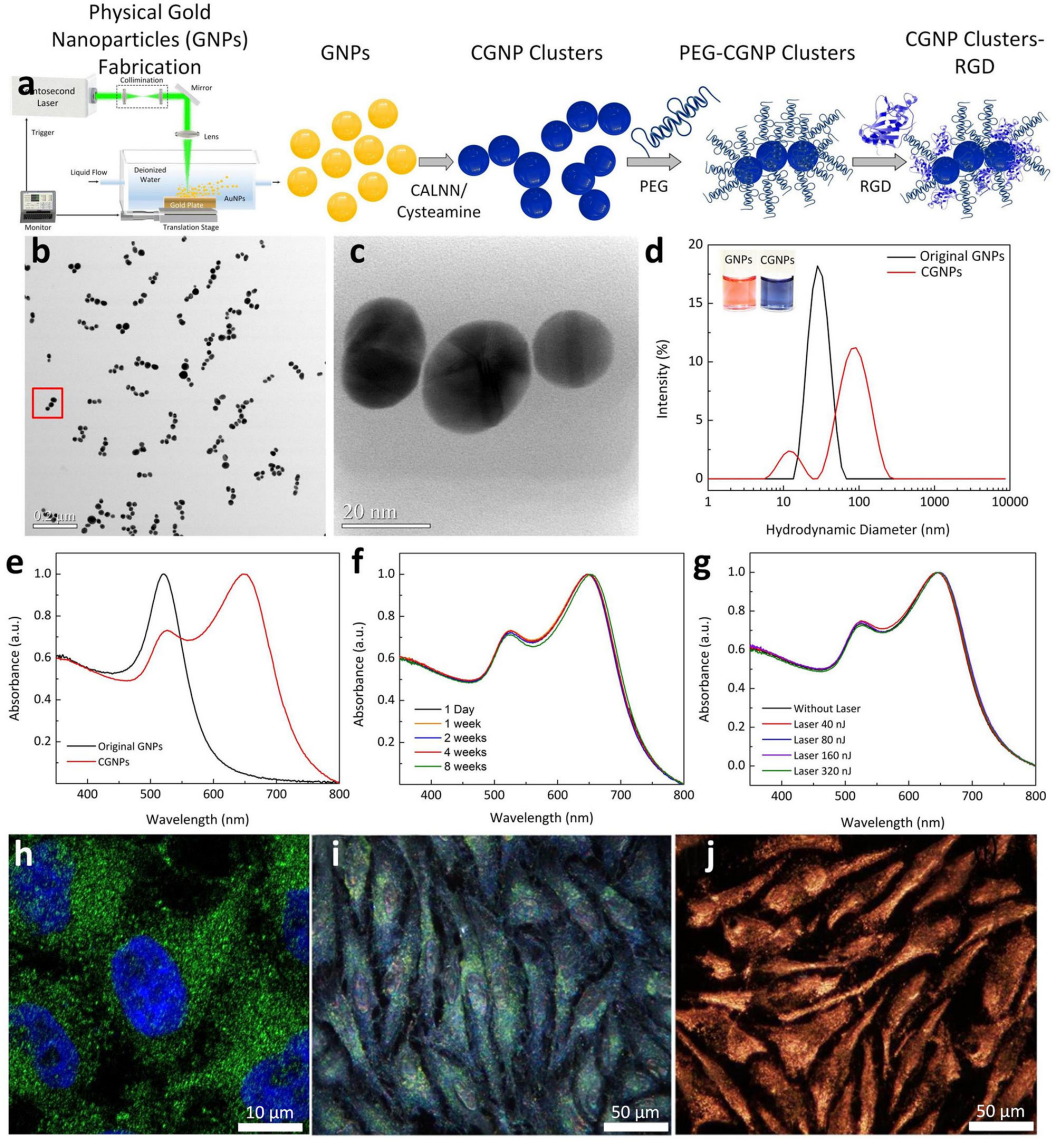
Characterization of synthesized chain-like gold nanoparticle clusters conjugated with RGD peptide (CGNP clusters-RGD) and quantification of the photoacoustic (PA) and optical coherence tomography (OCT) properties. **a** Illustration of synthesis of CGNP clusters-RGD. Ultra-pure colloidal gold nanoparticles (GNPs) were first fabricated by physical method (using femtosecond laser). Then, discrete GNPs were self-assembled using pentapeptide Cys-Ala-Leu-Asn-Asn (CALNN) and cysteamine in order to create CGNP clusters. **b** Transmission electron microscopy (TEM) image of CGNP clusters-RGD, ×2000, 20 kV; bar = 200 nm. **c** Region of CGNP clusters-RGD at high magnification, indicated by red square box in **b**. The CGNPs surrounded with a gray shell representing CALNN-PEG-RGD layer. **d** Comparisons of hydrodynamic size between GNPs and CGNP clusters-RGD measured in water by using dynamic light scattering (DLS). **e** UV-Vis absorption spectra of GNPs and CGNP clusters-RGD. **f** Quantitative characterization of the colloidal stability of CGNP clusters-RGD over time for up to 8 weeks by using UV-Vis absorption spectroscopy analysis. **g** In vitro photostability of CGNP clusters-RGD during nanosecond pulsed laser irradiation (65 s) at various pulse fluences (0.005, 0.01, 0.02, and 0.04 mJ/cm^2^). **h** Confocal fluorescent microscope image of single HeLa cell treated with FITC-labeled CGNP clusters-RGD at concentration of 50 µg/mL for 24 h. Blue fluorescent color indicates the cell’s nuclei stained with Hoechst 33342. Green color shows the position of CGNP clusters-RGD accumulated around the cell nuclei. **i** and **j** dark field images of HeLa cells after incubated with conventional GNPs-RGD with diameter of 20 nm and CGNP clusters-RGD, respectively. Note that the internalized CGNP clusters-RGD appeared indicating that CGNP clusters-RGD were stable in biological tissue. Source data are provided as a Source Data file.

Ultrapure colloidal solution of GNPs with an average diameter of 20 nm was fabricated by femtosecond pulsed laser ablation of gold target in DI water (Fig. 1a and see Supplementary Note 3). The self-assembly of GNPs into CGNP clusters in aqueous solution was performed by modifying GNP surface with two different types of ligands, pentapeptide Cys-Ala-Leu-Asn-Asn (CALNN) and cysteamine, in a sequential manner by first mixing the colloidal solution of GNPs with CALNN and then cysteamine. The CGNP clusters were then conjugated with polyethylene glycol (PEG) molecules and Arg-Gly-Asp (RGD) ligands (Fig. 1a). The transmission electron microscopy (TEM) images (Fig. 1b, c) revealed that the synthesized CGNP clusters-RGD had an average length of 64 nm and an average width of 20 nm determined by the diameter of the GNP. Their narrow size distribution was confirmed using dynamic light scattering analysis with polydispersity index of 0.3 (Fig. 1d). CGNP clusters-RGD had a redshift absorption peak at 650 nm (Fig. 1e) compared with that of GNPs. In addition, CGNP clusters-RGD had a large zeta potential of −41 ± 2 mV, which resulted in an excellent colloidal stability with a shelf life of longer than eight weeks (Fig. 1f). During 65 s of laser irradiations at various pulse fluences (i.e., 0.005, 0.01, 0.02, and 0.04 mJ/cm^2^), CGNP clusters-RGD exhibited high photostability at the wavelength of 650 nm (Fig. 1g). The Fourier transformed infrared spectroscopy (FTIR) was also used to confirm the presence of polyethylene glycol (PEG) on the surface of CGNP clusters-RGD. As it was demonstrated (see Supplementary Fig. S1), FTIR spectrum of CGNP clusters-RGD colloidal solution displayed all characteristic peaks that could be found in PEG with molecular weight of 2000. To examine the targeting specificity of CGNP clusters-RGD for the tissue of interest, confocal microscope and dark field microscope images were obtained for HeLa cells incubated with either RGD-conjugated GNPs with diameter of 20 nm or CGNP clusters-RGD (Fig. 1h–j). CGNP clusters-RGD were distributed within the cytoplasm of HeLa cells (green color) after incubating them with CGNP clusters-RGD (50 µg/mL) overnight, indicating that CGNP clusters-RGD effectively targeted and localized to the cancer cells (Fig. 1h). Dark field microscope image in Fig. 1j confirmed that CGNP clusters-RGD were stable within biological tissue because they appeared orange due to intense light scattering in the 650 nm range determined by their LSPR peak wavelength (disassembly of CGNP clusters-RGD would have been indicated by a shift in color to green as shown in Fig. 1i).

### In vitro contrast enhanced PAM and OCT of CGNPs

**Fig. 2:**
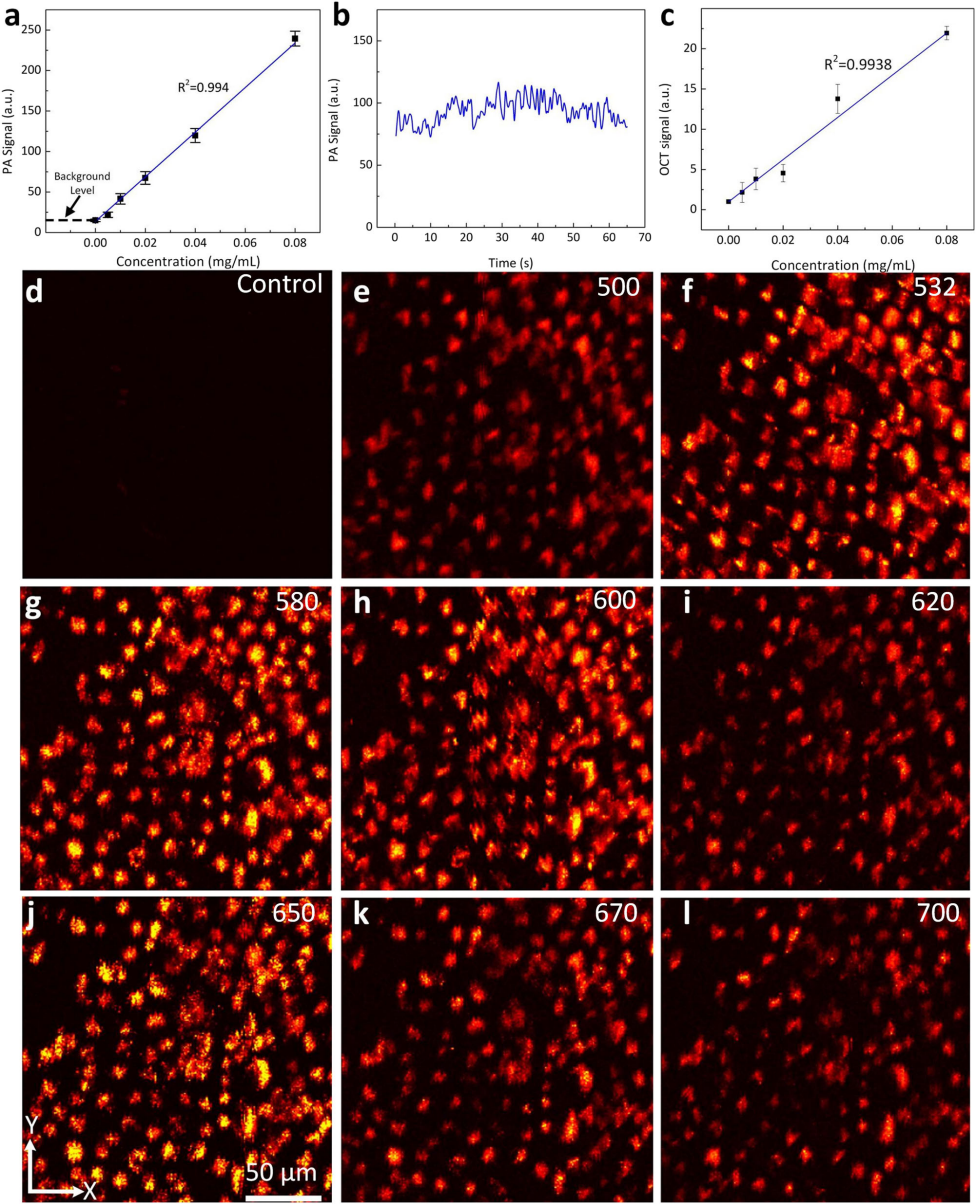
Quantification of the photoacoustic (PA) and optical coherence tomography (OCT) properties. **a** Quantitative PA signal of the CGNP clusters-RGD in phantom as a function of nanoparticle mass concentration and estimating the average PA amplitude above the background level in each image. (**b**) PA signal from CGNP clusters-RGD in phantom as a function of laser irradiation time. **c** Quantification of OCT signal from CGNP clusters-RGD of different concentrations in phantom. **d**–**l**) In vitro PAM images of cells acquired at various wavelengths ranging from 500 to 710 nm. The error bars in **a** and **c** represent the standard deviation of the mean PA signal measured from 3 different samples (*N* = 3). Source data are provided as a Source Data file.

The ability of CGNP clusters-RGD to enhance both PA and optical coherence tomography (OCT) signals was evaluated in order to determine the detection threshold for each modality. In a phantom study, silicone tubes were filled with solutions of CGNP clusters-RGD at different concentrations (i.e., 0 (saline), 0.005, 0.01, 0.02, 0.04, and 0.08 mg/mL) and imaged with a custom-built integrated photoacoustic microscopy (PAM) and OCT imaging system using light at 650 nm. It was shown that there was a linear relationship between the PA signals identified using a region of interest analysis (ROI) (see Supplementary Fig. S2) and the concentration of CGNP clusters-RGD in solution (Fig. 2a) (*R*^2^ = 0.9947). The lowest detectable concentration of CGNP clusters-RGD in solution by PA detection was estimated to be 0.01 mg/mL with a signal to noise ratio (SNR) of 1.9 dB. The detected PA signal was stable and did not vary more than 2% when illuminated with a nanosecond pulsed laser at a repetition rate of 1 KHz and laser fluence of 0.01 mJ/cm^2^ for a period of 65 s (Fig. 2b). The OCT signal generated by CGNP clusters-RGD (see Supplementary Fig. S3) similarly displayed a linear relationship with the concentration of CGNP clusters-RGD (*R*^2^ = 0.9938) (Fig. 2c). The lowest detectable concentration by OCT was 0.005 mg/mL.

The ability of CGNP clusters-RGD to enhance the PA signal was first examined using HeLa cells. HeLa cells with internalized CGNP clusters-RGD were imaged with a custom-built PAM system, and the PA spectrum was acquired as a function of wavelength ranging from 500 to 710 nm. HeLa cells not treated with CGNP clusters-RGD displayed no contrast (the detected PA signal came from the background noise floor, Fig. 2d). In contrast, HeLa cells with internalized CCNP clusters-RGD generated a strong SNR with an average of 14.45 dB at the excitation wavelength of 650 nm (PA_signal_ = 152.56 ± 18.71 (a.u) for cells vs. PA_signal_ = 5.47 ± 0.50 (a.u.) for background) (Fig. 2e–i). Quantitative PA spectrum of HeLa cells labeled with CGNPs shows correlation with the absorption spectrum determine by UV-Vis spectrometry displayed on Fig. 1e, with a peak occurred at 650 nm due to redshift plasmonic peak of CGNPs (see Supplementary Fig. S1b).

### In vivo biosafety and biodistribution of CGNPs

The toxicity of CGNP clusters-RGD was determined by evaluating whether CGNP clusters-RGD could induce undesired side effects in vitro and in living animals. An in vitro toxicity study showed that CGNP clusters-RGD only caused a slight decrease in cell viability for various cell types, including macrophage cells (Raw.467), cancer cells (HeLa), bovine brain endothelial cells (b.End3), and bovine retinal endothelial (BREC) cells. The percentage of cell survival was more than 80% even at a long incubation time of 48 h using high concentration of CGNP clusters-RGD (500 µg/mL) (see Supplementary Fig. S4a–S4d). Next, flow cytometry analysis was used to investigate the potential of CGNP clusters-RGD to induce apoptosis (see Supplementary Fig. S4e–4n). A quantitative viability comparison between negative control cells and CGNP clusters-RGD-treated cells highlighted a slight decrease in the viable cell populations at incubation times of 24 and 48 h. For example, after 48 h, the number of living, apoptotic, and necrotic cells over the total number of cells were 95.17 to 85.96%, 0.18 to 5.62%, and 2.47 to 10.85%, respectively, indicating that CGNP clusters-RGD were nontoxic to the cells (see Supplementary Fig. S4o–p). These results were consistent with the conclusions from the confocal fluorescence imaging (see Supplementary Fig. S5).

To investigate the biosafety of CGNP clusters-RGD in vivo, mice were administered with either conventional colloidal GNPs or CGNP clusters-RGD of various amounts (2.5, 5, 10, and 20 mg/kg) for 7 days (*N* = 3 per condition). The body weight of each mouse was then monitored for the subsequent week. As shown in Supplementary Fig. 6a–b, the treated mice had regularly body weight proliferated during 7 days after each medication condition, indicating that both GNPs and CGNP clusters-RGD did not induce toxicity or lethality in the groups.

Histological analyses were performed to evaluate the effect of CGNP clusters-RGD on tissues in vivo. The hematoxylin and eosin images showed normal cellular morphology, and TUNEL images displayed no cells undergoing apoptosis with CGNP clusters-RGD (see Supplementary Figs. S7 and S8). The acute toxicity of CGNP clusters-RGD was then assessed using kidney and liver function tests. Over the period of the 14-day study, there were no adverse changes in the rabbits’ alanine amino transferase (ALT), blood urea nitrogen (BUN), or alkaline phosphatase (ALP) levels (Table 1), indicating no systemic, kidney, or liver toxicity. This is notable since both conventional GNPs-RGD and CGNP clusters-RGD accumulate primarily in the liver and spleen as demonstrated from biodistribution studies. Conventional GNPs-RGD showed a 4.10-fold and 2.36-fold higher accumulation in the spleen than that of CGNP clusters-RGD at a concentration of 10 and 20 mg/kg, respectively (see Supplementary Fig. S9a). Similar findings were reported in a recent study published by Glazer et al.^13^ that demonstrated larger GNPs had less accumulation in the spleen compared with smaller GNPs. Finally, to determine the circulation time of CGNP clusters-RGD in living rabbits, colloidal solution of CGNP clusters-RGD at two different concentrations (i.e., 2 and 4 mg/mL) were intravenously injected via the rabbit marginal ear vein. Blood samples were then collected at various time points ranging from 15 min to 72 h. It was found that the amount of CGNP clusters-RGD in circulation gradually decreased for both concentrations, and the half-life in circulation was estimated to be 30 min (see Supplementary Fig. S9b).

**Table 1:** Liver and kidney function tests in rabbits.

Test	Unit	Normal range	Control 1	Control 2	Control 3	Treated 1	Treated 2	Treated 3
Creatinine	mg/dL	0.5–2.6	1.08	1.21	1.11	1.06	0.79	1.05
Albumin	g/dL	2.7–5	4.1	4.3	4.0	4.5	3.7	3.6
Total protein	g/dL	5–7.5	5.8	6.6	6.2	7.1	5.8	5.4
Alanine transaminase	U/L	25–65	32	44	54	36	40	56
Total bilirubin	mg/dL	0.2–0.5	0.05	0.09	0.08	1.76	0.04	0.09
Blood urea nitrogen	mg/dL	5–25	19	18	17	20	19	14
Alkaline phosphatase	U/L	10–86	82	83	79	81	83	80

### Multimodal molecular PAM and OCT imaging system

**Fig. 3:**
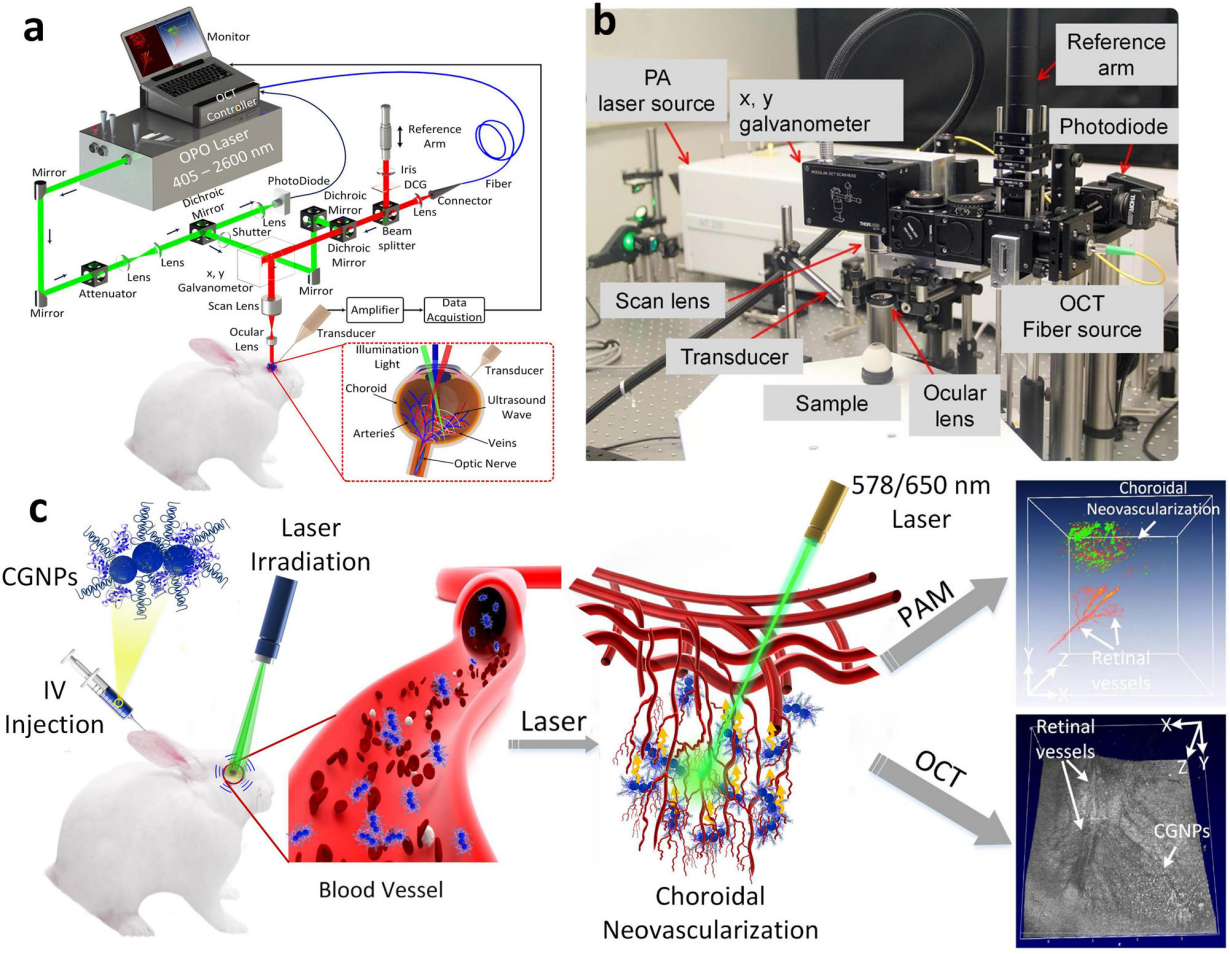
Experimental setup of multimodal PAM and OCT equipment. **a** Schematic diagram of the imaging system. **b** Physical setup. In PAM mode, nanosecond excitation laser with tunable wavelength output in 450–710 nm range was delivered and focused onto the retina. In addition, to enable multimodal imaging, the excitation laser beam used to induce PA signal was coaxially aligned with OCT multispectral luminescence with a center wavelength at 805 nm and 905 nm (OCT). A needle-shaped hydrophone ultrasonic transducer was used to detect the generated acoustic signal and then, the recorded data was utilized to reconstruct PAM images. The reflected OCT light interfered with the reference light and the interference intensity spectra was detected by a spectrometer. As the retina was scanned by a galvanometer, the acquisition time is approximately 65 s for a field of view of 4 × 4 mm with a resolution of 256 × 256 pixels and the 3D volumetric visualization was rendered. **c** Illustration of the in vivo multimodal imaging using CGNP clusters-RGD. The synthesized CGNP clusters-RGD were intravenously injected into the rabbit model through marginal ear vein. PA signals from the rabbit retina was generated by using nanosecond pulsed laser illumination at wavelength of either 578 or 650 nm.

Unlike most PA ocular imaging systems for the eye, which are used to visualize the retinal vessels and retinal tissues (RPE) of small animals (mice and rats)^14–18^, the current study developed a multimodal imaging system employed for larger animals. This is an important step to transfer this technique to clinical application because the axial length of the rabbit eye and the human is very similar (i.e., axial length = 18 mm for the rabbit eye vs. 23 mm for the human eye), whereas the rat and mouse axial length is 6 and 3 mm, respectively. The custom-built imaging system provides several advantages, particularly in relation to partial resolution and acquisition time, resulting in improved visualization of individual capillaries and neovascularization in large animal eyes when compared with conventional ocular PA imaging. Figure 3a, b illustrates a schematic and physical setup of the multimodal imaging system including PAM and OCT. The current system has excellent axial and lateral resolution due to its use of focused light and the custom-made ultrasound transducer. The quantified lateral resolution of PAM and OCT are 4.1 and 3.8 µm, respectively. The axial resolutions are 37.0 µm for PAM and 4.0 µm for OCT. With the high resolution, this system is sufficient to detect microvasculature, capillaries, and newly developed neovascularization, which has overcome the drawback of the current photoacoustic ocular imaging systems that is only capable of visualizing the large retinal blood vessels of the medullary ray. In addition, the acquisition time is also suitable to achieve high-resolution PAM image. To achieve a volumetric image of 4 × 4 mm, the acquisition time is approximately 65 s with a resolution of 256 × 256 pixels.

### Longitudinal multimodal PAM and OCT visualization of choroidal neovascularization in living rabbits

**Fig. 4:**
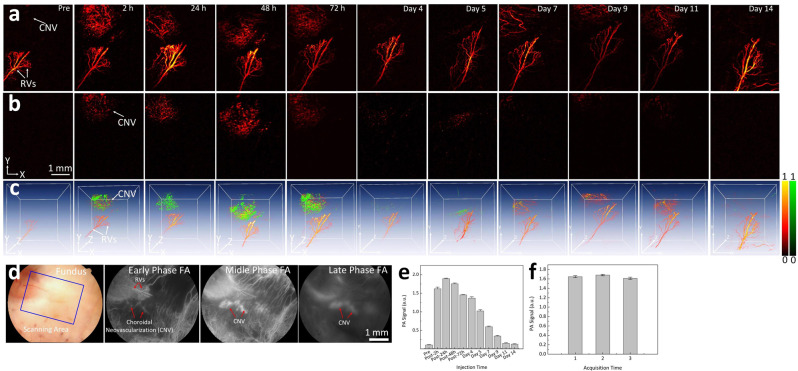
PA detection of CNV in living rabbits using CGNP clusters-RGD. **a** and **b** PAM images of CNV before and after the injection of 0.5 mL CGNP clusters-RGD at concentration of 2.5 mg/mL acquired along the selected area outlined in fundus image **d** under nanosecond pulsed laser illumination at wavelength of 578 and 650 nm, respectively. **c** Overlay 3D images showed the distribution of CGNP clusters-RGD accumulated at CNV location in rabbit retina (pseudo-green color). **e** Rabbit injected with CGNP clusters-RGD exhibited significantly higher PA signal than pre-injection. Note that the peak PA signal occurred at 24 h post injection. Then, the PA signals gradually decreased over time. **f** In vivo photostability of CGNP clusters-RGD. The error bars in **e** and **f** represent standard error of the average PA signal measured from three different animals (*N* = 3), *p* < 0.05. Source data are provided as a Source Data file.

**Fig. 5:**
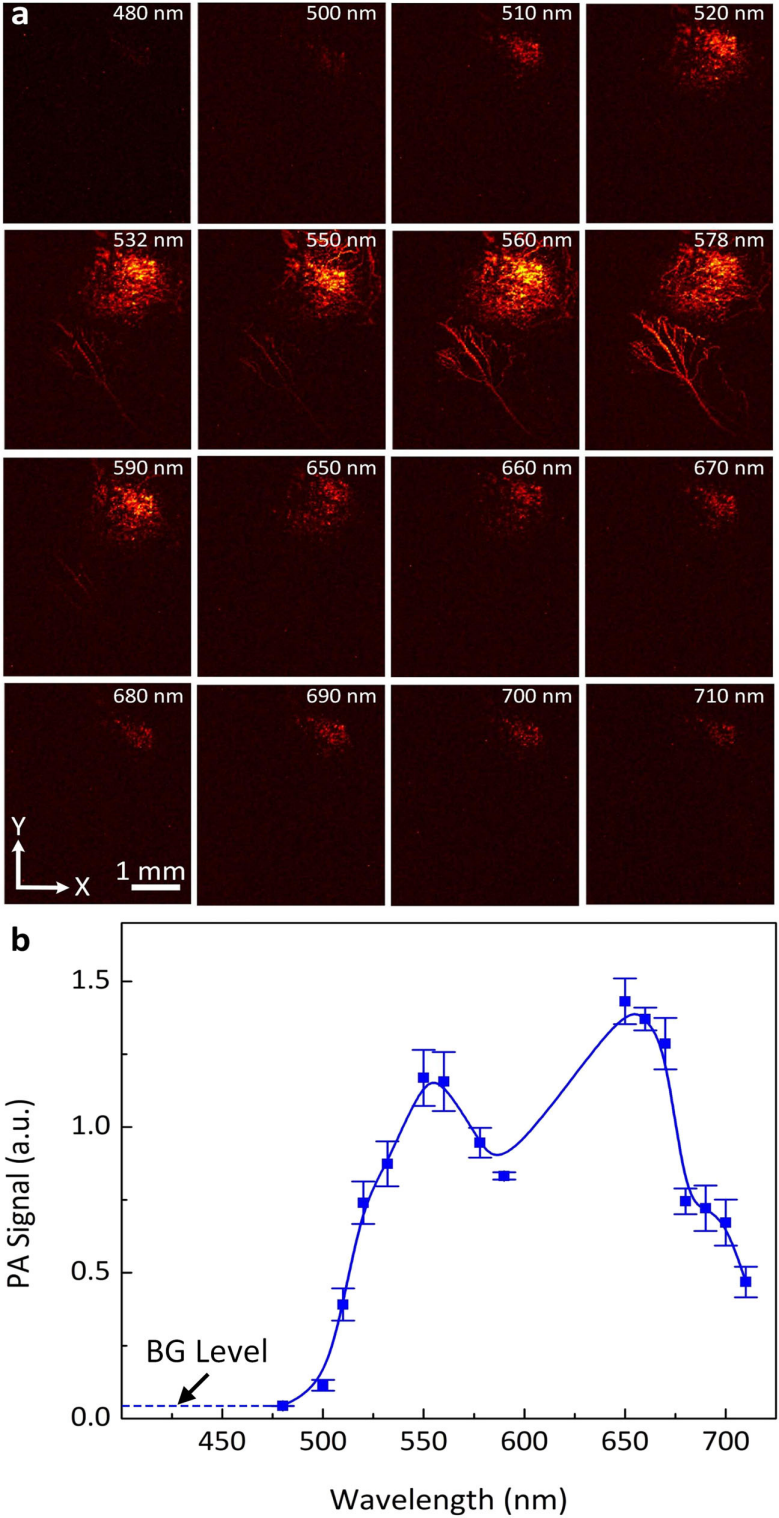
In vivo spectroscopic PAM imaging of CNV. **a** Spectroscopic PAM images acquired at the different excitation laser wavelengths ranging from 480 to 710 nm. **b** Quantitative measurement of spectroscopic PA signal as a function of wavelength. BG background. Source data are provided as a Source Data file.

CGNP clusters-RGD were utilized to detect CNV in living rabbits (Fig. 3c). It was hypothesized that CGNP clusters-RGD would target α_v_β_3_ integrin, which is expressed in CNV but not in the normal vasculature. Two-dimensional (2D) and three-dimensional (3D) control PA images were employed at dual wavelength of 578 nm (Fig. 4a) and 650 nm (Fig. 4b) prior to CGNP clusters-RGD administration. A group of laser-induced CNV rabbits (*N* = 3) were injected with 0.4 mL (5 mg/mL) of CGNP clusters-RGD through the marginal ear vein. Both 2D and 3D PA images of the CNV and the surroundings were obtained at 2 h, 24 h, 48 h, 72 h, 5, 7, 9, 11, and 14 days after injection (Fig. 4 and Supplementary Fig. S10). Post injection of CGNP clusters-RGD, images were acquired along the selected areas shown in Fig. 4d using light with a wavelength at 650 nm allowing easy visualization of CNV, where no contrast enhancement had been observed pre-injection. The PAM images of CNV and surrounding vasculature acquired at both 578 and 650 nm exhibited a significant increase in PA signal compared with the pre-injection images. The position of CNV was clearly distinguished from retinal vessels using an excitation wavelength of 650 nm at which the optical absorption by blood vessels is minimal (Fig. 4b). In contrast, there was not a significant increase in PA signal acquired at 650 nm after injection of CGNPs without conjugation to RGD ligands (Supplementary Fig. S11a–c). Figure 4c shows a 3D volumetric visualization of the data set obtained from two different wavelengths and co-registered in same orthogonal planes using Amira software (see Supplementary Movie 1 and Supplementary Note 7). These images exhibit notable contrast and strong optical absorption of CGNP clusters-RGD against the adjacent vasculature. We calculated the PA signals by selecting a ROI around the CNV. The quantification of PA signals in the CNV as a function of time showed a significant increase in PA signals compared to images acquired before intravenous administration (Fig. 4e). There was a 17-fold increase from 0.11 ± 0.01 to 1.89 ± 0.1 from the peak PA signal obtained at 24 h after injection of CGNP clusters-RGD (*p* = 0.001). In vivo, the photostability of CGNP clusters-RGD was characterized by quantifying the PA signal from the ROI at different scanning times (Fig. 4f and Supplementary Fig. S12). We found that the PA signals did not vary more than 2% (i.e., 1.64 ± 0.02, 1.68 ± 0.02, and 1.61 ± 0.02 (a.u.) for the first, second, and third scan, respectively). PA signals at wavelengths ranging from 480 to 710 nm were then acquired to determine the spectroscopy PA signal (Fig. 5). The ability to achieve an even greater contrast between healthy blood vessels and CNV by using longer excitation wavelengths is shown in Fig. 5a. The spectroscopic data (Fig. 5b) indicated that the peak PA signal occurred at 650 nm, which is consistent with the absorbance spectra shown in Fig. 1e. The diameter of the larger and smaller retinal vessels was estimated to be 70.84 ± 15.26 and 30.26 ± 5.01 µm, respectively. The detected area of CNV was measured to be 1.70 ± 0.07 mm^2^ (Supplementary Fig. S13).

**Fig. 6:**
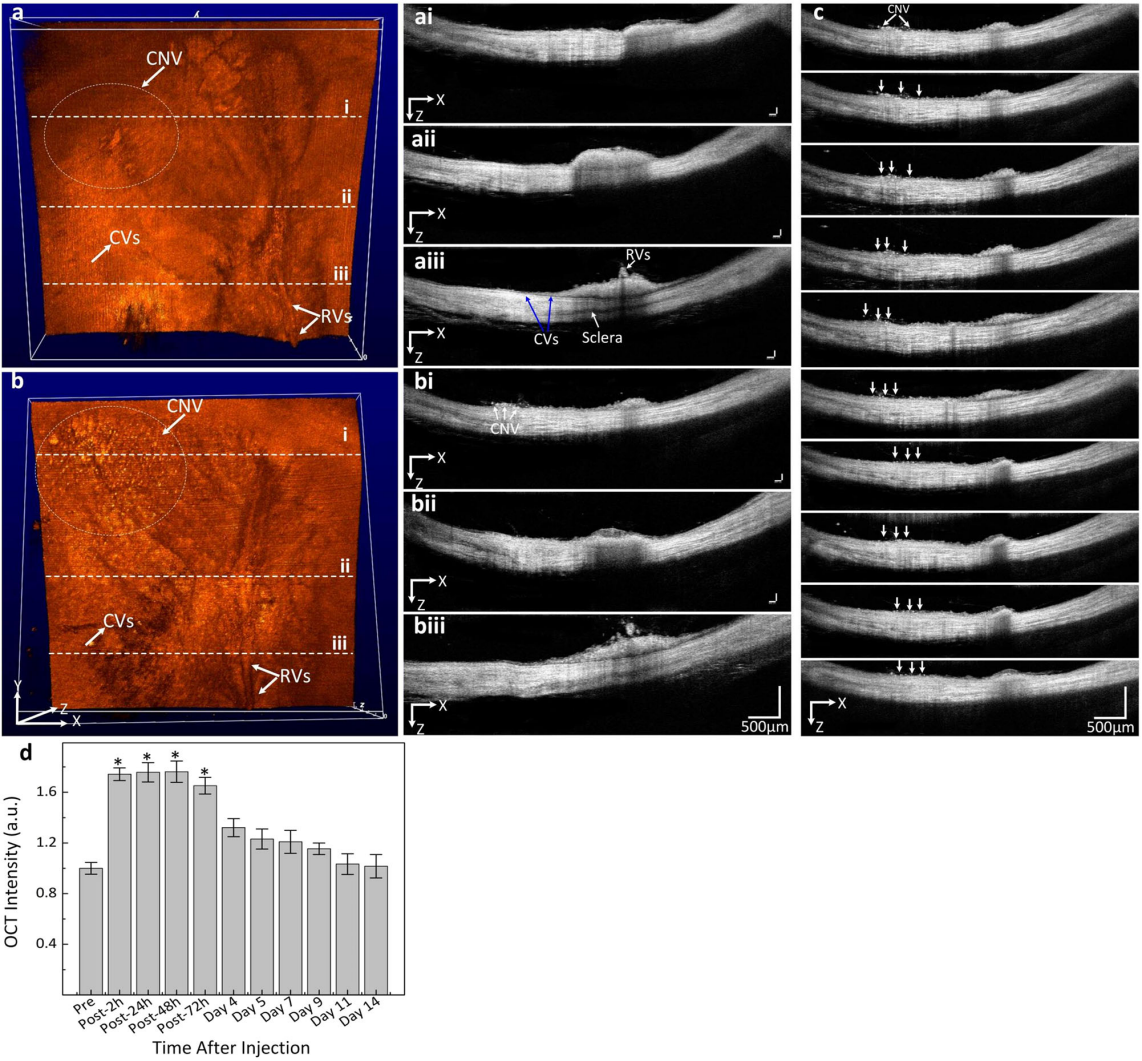
In vivo contrast enhancement of OCT images of CNV in rabbits. **a**, **b** 3D OCT images before and after the injection of CGNP clusters-RGD. **ai**–**aiii**, **bi**–**biii** B-scan OCT images of one vertical slice (white dotted lines) through the retina. The distribution of CGNP clusters-RGD were clearly observed on 3D OCT image after injection (white dotted circle). **c** Cross-sectional B-scan OCT images obtained at different time points after the administration of CGNP clusters-RGD (2 h, 24 h, 48 h, 73 h, days 4, 5, 7, 9, 11, and 14). White arrows show the position of CNV. Rabbit injected with CGNP clusters-RGD show significant enhancement of OCT signals. **d** The error bars in **d** represent the standard deviation of the mean OCT intensities (*N* = 3). Source data are provided as a Source Data file.

**Fig. 7:**
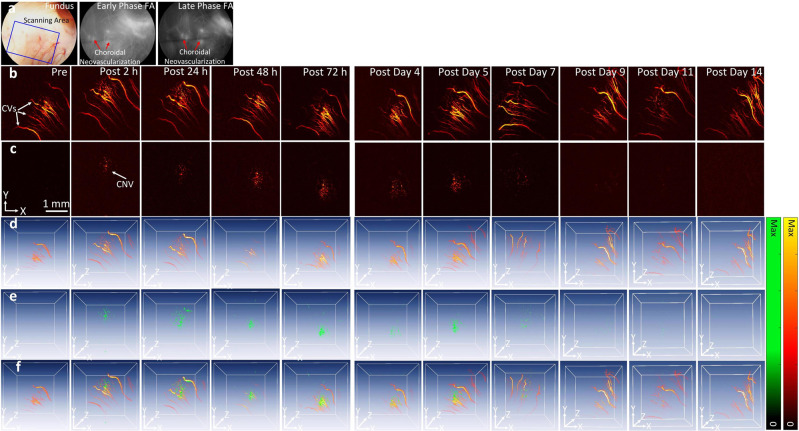
Another study of contrast-enhanced PA detection of CNV model in living rabbits. **a** Color fundus photography and fluorescein angiography images of the retina before injection of CGNP clusters-RGD. **b**, **c** PAM images of CNV acquired at 578 and 650 nm before and after the injection of CGNP clusters-RGD (0.5 mL at concentration of 2.5 mg/mL). **d** Fused 3D visualization shown in **b** and **c**. The retinal vasculature is shown in red and the distribution of CGNP clusters-RGD is shown in green. **e** 3D volumetric visualization of the PAM data acquired at 578 and 650 nm. **f** Fused 3D visualization shown in **d** and **e**.

OCT images of the rabbits were also obtained. Figure 6 shows the ability of our multimodal system to detect CGNP clusters-RGD after IV injection. Cross-sectional B-scan and conventional 3D volumetric OCT images were acquired at different time points. Spectral contrasts were measured by selecting the ROI on the B-scan images. Figure 6a illustrates the initial OCT signal of retinal blood vessels and CNV. Following the administration of CGNP clusters-RGD, a significant increase in OCT signal from the CNV was observed (Fig. 6b). Figure 6c shows the B-scan OCT images acquired at different time points after the administration of CGNP clusters-RGD (2 h, 24 h, 48 h, 73 h, days 4, 5, 7, 9, 11, and 14). The location of CNV was clearly observed at 2 h post injection and reached peak at 48 h. Then, the OCT signal gradually reduced over time. A ROI analysis was performed to quantify the significant increase in the OCT signal from the CNV (Supplementary Fig. S14) after the intravenous injection of CGNP clusters-RGD. The OCT contrast to noise ratio (CNR) increased from 1 to 1.76 (Fig. 6d). The capability of CGNP clusters-RGD for enhancing PA signal was verified on another rabbit model to confirm the repeatability of the agents. CNV was also created using laser photocoagulation in the presence of Rose Bengal as described previously^19–21^. The PAM images were acquired along the location of CNV beneath the optic nerve (Fig. 7a) at the excitation wavelength of 578 nm (Fig. 7b) and 650 nm (Fig. 7c). CNV was clearly visualized and distinguished after injection of CGNP clusters-RGD (Fig. 7c–f). Both 2D and 3D images were reconstructed from the raw data acquired by the ultrasound transducer without any post image processing. In addition, 3D volumetric PAM images identified the volume and depth of blood vessels.

**Fig. 8:**
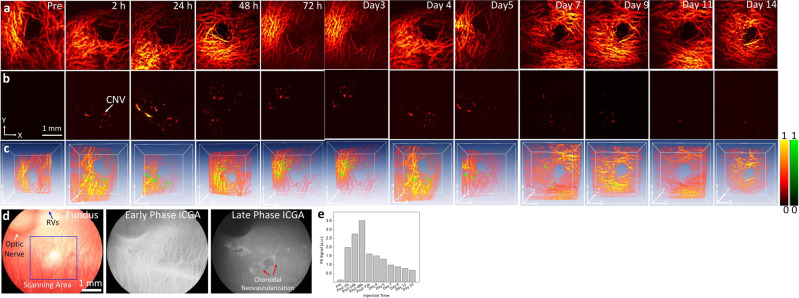
Study of contrast-enhanced PA detection of CNV model in living rabbits induced by sub-retinal injection. **a**, **b** PAM images of CNV obtained along the selected region shown in color fundus image **d** at 578 and 650 nm, respectively, before and after the injection of CGNP clusters-RGD (0.5 mL at concentration of 2.5 mg/mL). The CNV was obviously distinguished at 650 nm. **d** Fused 3D visualization of the data set shown in **b** and **c**. The retinal vasculature is shown in red and the distribution of CGNP clusters-RGD is shown in green. The color bar represents the normalized image intensities of the control (red) and CGNP clusters-RGD (green). The volumetric acquisition time was approximately 65 s. **e** PA signals measured for each time points. The error bars represent the standard deviation of the mean PA signals (*N* = 3). Source data are provided as a Source Data file.

Rabbits with CNV created by subretinal release of mixed Matrigel and VEGF-165 similarly underwent optical imaging. PAM images were captured along the selected regions shown in Fig. 8d at multiple wavelengths (i.e., 578 and 650 nm) for both pre-injection and 2, 24, 48, 72 h and days 4, 5, 7, 9, 11, and 14 post injection (Fig. 8a–c). When compared to the pre-injection images, it was found that the PA signal was significantly enhanced 2 h post injection (from 0.13 ± 0.001 to 1.97 ± 0.02 (a.u.) for pre- and post-administration, respectively; *p* < 0.001) due to the specific targeting of CGNP clusters-RGD to the CNV microvasculature (Fig. 8e). The PA signal reached a maximum at 48 h before gradually decreasing and remaining stable from day 7 to day 14. Compared to the pre-injection data, the CGNP clusters-RGD provided PA signal enhancement of higher than 26 times at 48 h post injection. To reveal the clear improvements of CGNP clusters conjugated with RGD targeting ligands for contrast-enhanced PAM, we compared the PAM signals of CGNP clusters-RGD and CGNP clusters without RGD. It was found that no PAM signal was detected on PAM images acquired at 650 nm pre- and post injection of CGNPs cluster only (Supplementary Fig. S11d–f). Thus, CGNP clusters-RGD could be very useful as diagnostic agents for the detection of CNV due to their strong optical absorption and scattering in the red/NIR region of the spectrum where blood vessels generate less signal.

## Discussion

This study described ultra-pure CGNP clusters that can be used as multimodal PAM and OCT contrast agents. These synthesized agents demonstrated excellent biocompatibility and photostability and were tested in vivo to image choroidal microvasculature in living rabbits, a clinically relevant animal model. Intravenous injections of CGNP clusters-RGD in rabbits with CNV led to a 17-fold increase in PA amplitude and an increase in OCT amplitude by 176% compared to pre-injection. CNV was clearly discernible when acquiring PAM images at multiple wavelengths, and the CGNP clusters-RGD did not accumulate in the surrounding vasculature. Thus, targeted CGNP clusters-RGD could facilitate the ability to visualize both the location and margin of CNV in 3D, allowing for early detection and precise CNV treatment.

The fabricated CGNP clusters have an excellent advantage over conventional colloidal GNPs. CGNP clusters have been designed to shift the LSPR peak wavelength from 520 nm to the red/NIR region of the spectrum (e.g., 650 nm), while maintaining the original size of the GNPs at the smallest diameter of 20 nm. This is different from previous studies, which use large gold nanoparticles as contrast agents for PA or OCT imaging^9^. Such studies have reported that the peak wavelength of colloidal gold nanoparticle was shifted to 650 nm at a large diameter of ~100–150 nm or using gold nanorods with the size of 30 × 100 nm^2,^^22^. However, a major drawback is their relatively large size, which may cause them to be rapidly cleared from the body via the RES or have long-term toxic effects. Alkilany et al. reported that large GNPs could have high toxicity at the tested concentrations in mice, cell cultures, and a number of clinical trials^8^. In contrast, other studies reported by Haute and Chou et al. reported that reducing the size of GNPs could avoid long-term toxicity and found that the renal clearance threshold was less than 5.0 nm^23–25^. In addition, with a higher surface–volume ratio, the PAM signal could be further enhanced, as demonstrated in a recent work published by Chen et al.^26^ The authors demonstrated that small gold nanorods (GNRs) (i.e., width = 8 nm) can increase the PA signal by more than 3.5-fold when compared to larger GNRs (i.e., width = 17 nm).

CGNP clusters not only demonstrated good stability in physiological environments in the short term, excellent photostability, and non-toxic at both cellular levels and in vivo at tested concentrations but also were disassembled in living beings after 1–4 weeks post injection (Supplementary Fig. S15), resulting in improved clearance and likely avoidance of long-term toxicity. This finding is consistent with a recent reported by Li et al. The authors have shown that GNPs at a size of about 24.3 nm could be excreted in less than 30 days^27^. Based on strong optical absorption and scattering properties in the red/NIR region of the spectrum, CGNP clusters are suitable for CNV imaging using PAM and OCT. A concentration of 0.67 mg/kg of CGNP clusters-RGD was found to generate a strong PA signal at 650 nm, while hemoglobin produced a very low intrinsic PA signal at that wavelength, making it feasible to discriminate the distribution of the exogenous contrast agent from the background (normal vasculature). More importantly, in vivo experiments were performed with a single injection of the CGNP clusters-RGD and showed that the CGNP clusters accumulated and stayed in the CNV for up to 5 days, allowing for repeated imaging without requiring multiple injections. This finding is different from previous studies using GNPs for PA and OCT imaging. Liba et al. showed that large GNRs had a peak OCT contrast 2 h post injection and the OCT signal was significantly reduced at 24 h^2^. Similarly, Jing et al. reported that the peak PA signal using GNPs occurred 22 h post injection^11^. The limitation of these studies is that they used nanoparticles without targeting a specific molecular peptide, resulting in rapid clearance from the body. It is similar to the experimental data shown in Supplementary Fig. S11 using CGNPs cluster without conjugation with RGD ligand. There is no detectable PAM signal from the PAM image after the injection of CGNP clusters. Another advantage of CGNP clusters is the higher molecular weight compared to organic fluorochromes such as ICG, Prussian Blue, or Methylene Blue. Nanoparticles made using these fluorochromes having low molecular weight will be rapidly cleared from the body and not be able to target the disease sites of interest. As a result, multiple doses of these nanoparticles are required to enhance the PA signal.

Another finding is that the kinetics of signal enhancement is different between the PA and the OCT imaging after administration of nanoparticles as shown in Figs. 4e and 6d. We hypothesize that this is because the PAM signal is based on plasmonic absorption of nanoparticle with a peak absorption at 650 nm where hemoglobin produces very low PA signal at that wavelength. In addition, we also found in our pervious study that signal enhancement did not always directly correlate between OCT and PAM imaging and that this is an area of active investigation of our group^20^. This could be due to the difference in relative signal intensity from the two different circulations present in the eye, the retinal, and choroidal circulation. The choroidal circulation departs from the ophthalmic artery earlier and thus the choroidal flush appears earlier than the retinal circulation on fluorescein angiography.

Furthermore, a major challenge in ophthalmology is the lack of an advanced imaging tool to detect newly developed blood vessels in the sub-RPE space of the retina. While OCT and OCTA are widely applied to visualize healthy and abnormal vessels such as retinal blood vessels, RVO, and RNV with high resolution and high contrast, OCT and OCTA images are created from backscattering light in target tissue, limiting their penetration depth^28^. In addition, angiographic imaging can be created using OCTA by performing motion contrast imaging to volumetric blood flow information. OCTA repeatedly scans the same discrete tissue volume and detects changes in the reflectivity signal. It is difficult to avoid motion artifacts in OCTA imaging as a result of the movement of blood flow in the back of eyes. The minimum detectable flow rate is determined by the time between two consecutive B-scans on OCT, and thus OCTA can miss some areas of slow blood flow. This is very important clinically because slow blood flow is of particular interest to clinicians, namely visualization of leakage from angiogenesis and microaneurysms. OCT and OCTA can achieve high-resolution imaging of retinal structure and some functional information, but they have a limited identification of the choroid and limited capabilities to perform molecular imaging due to the high background signal for molecular imaging. The seeing neurons called photoreceptors receive their blood supply with a deeper blood vessel supply called the choroid particularly in the central vision, the fovea, where there is a foveal retinal avascular zone. OCT, OCTA, and other optical only imaging techniques have difficulty penetrating to this depth to visualize with high resolution the choroidal vessels and instead report a measurement of choroidal thickness, which serves as a limited biomarker for disease. Thus, the current study with integrated OCT and PAM overcomes the limitation of OCT and better observes the retinal vascular network and newly developed choroidal neovascularization. Structural and functional information of the retinal vasculature was quantitatively identified using spectroscopic PAM. The spectroscopic PA images were achieved pre- and post injection of contrast agents at various wavelengths, which will lead to the selection of optimal excitation wavelength for achieving maximum PA image contrast and improved CNV classification capacity. In addition, the registration of multiple PA images acquired at different wavelengths on the same orthogonal imaging plane can help to avoid systemic errors such as image segmentation or background subtraction processing. Particularly, the developed multimodal imaging system used in this study provides excellent lateral and axial resolution, permitting discrimination and identification of the pathogenesis of choroidal neovascularization.

Several multimodal photoacoustic systems for visualization of retinal tissues have been developed to visualize structural and functional information of the retina, including the retinal pigment epithelium, melanin, retinal neovascularization, corneal neovascularization, and choroidal neovascularization with promising results^29^. A major challenge is that most of these studies have been performed on small animal eyes, such as mice and rats in which the axial length of the eye is much smaller than that of the axial length of human eyes (i.e., ~3 mm for mice, ~ 6 mm for rats vs. ~18 mm for rabbits and ~23 mm for human), limiting their possible clinical translation. Our system was developed to visualize the retinal structures in larger animals. In previous studies, our group has shown that the multimodal imaging system could achieve label-free PAM and OCT images of individual retinal vessels, choroidal vessels, retinal neovascularization, and retinal vein occlusion in rabbit with high resolution and high image contrast. In addition, with high axial and lateral resolution, our system can visualize individual single cells with a resolution comparable with commercial confocal microscopes as shown in Fig. 2e–l.

Although the current system can detect and monitor the development of newly developed blood vessels in the sub-RPE space of the retina with high resolution and high PAM image contrast, there are several improvements that need to be addressed before translating this technique clinically. First, the acquisition time of this current system will need to be improved since high imaging speed is mandatory in photoacoustic imaging in the eye to avoid potential motion artifacts. Robinson et al. have described the fixation time of the eye as approximately 500 ms^30^. Motion artifact can induce image disruption or image blurring. The scanning time is limited by the laser repetition rate of 1 kHz of the optical parametric oscillator (OPO), but the current scanning time can readily be increased by switching to a high-speed laser source that is not tunable. For example, the acquisition time can be decreased to roughly 6.5 s by using a 10 MHz nanosecond pulse duration single wavelength laser illumination that is commercially available.

In summary, this study presented a biocompatible, photostable, and ultra-pure CGNP clusters conjugated with RGD peptides for multimodal PAM and OCT contrast agents. These CGNP clusters-RGD are validated for multimodal PAM and OCT imaging of CNV in vivo in rabbits. The CGNP clusters-RGD exhibiting a redshift absorption peak at 650 nm, were remarkably stable in physiological environments, biocompatible, and could specifically bind to molecular targets for a significant time. The CGNP clusters-RGD were photostable under nanosecond pulsed laser illumination and produced a stable photoacoustic signal, which is critical for achieving quantitative PAM imaging and reducing the ambiguous evaluation of extravasation contrast agent concentrations. Due to strong plasmonic-enhanced optical absorption and scattering, even at low doses, CGNP clusters-RGD significantly enhanced both PA and OCT signal and completely differentiated CNV from peripheral retinal microvasculature. The multimodal PAM and OCT imaging using the CGNP clusters provided high-contrast, low-background, and 3D volumetric CNV anatomy and function with depth information and sub-micrometer resolution. Future work could include further quantification of the degree of extravasation of the CGNP clusters from tissue and surface modification of CGNP clusters for theranostic purposes such as targeted drug delivery or locally activated targeted laser therapy. In addition, the diameter of GNPs used for the fabrication of CGNP clusters could be further reduced to allow better clearance from the body and to minimize the long-term toxicity^23^. This work will open a new avenue for targeted multimodal molecular imaging.

## Methods

### Synthesis of CGNP clusters-RGD for CNV targeting

CGNP clusters-RGD were fabricated as a multimodal exogenous contrast agent for PAM and OCT imaging. Briefly, ultrapure GNP monomers were produced by the method of pulsed laser ablation (PLA) developed in IMRA America, Inc., which involves only bulk gold target (purity = 99.99%) immersed in 18 MegOhm DI water and a laser beam at 1045 nm from an ytterbium-doped femtosecond fiber laser (FCPA μJewel D-1000, IMRA America, Ann Arbor, MI) (see Supplementary Notes 1 and 2). Unlike typical GNPs fabricated by chemical methods that requires chemical precursors, reducing agents, and stabilizers, PLA-generated GNPs are naturally negatively charged and no capping agents/stabilizing ligands are required for maintaining their colloidal stability, which leads to minimal impurities^31^. The self-assembly of spherical GNPs into CGNP clusters was performed by modifying the surface of GNPs with two different types of ligands, pentapeptide with an amino acid sequence of CALNN and cysteamine, in a sequential manner by first mixing the colloidal GNPs with CALNN and then cysteamine. Colloidal GNPs with an average diameter of 20 nm were mixed with a solution of CALNN peptides to achieve a defined molar ratio of 2000:1 between CALNN peptides and GNPs. The mixture of GNPs and CALNN peptides was kept undisturbed for 2 h at room temperature to enable sufficient conjugation of CALNN peptides to the GNPs via Au-sulfur bonds. Then, surface of GNPs was further modified with cysteamine molecules by mixing with cysteamine solution to achieve a molar ratio of 1800:1 between cysteamine molecules to GNPs. The solution was kept undisturbed at room temperature for 24 h or several days after addition of cysteamine molecules until the self-assembly of GNPs into one-dimensional CGNP clusters was completed from the observation of an obvious color change from red-pink to blue. The raw CGNP clusters were then functionalized with both PEG molecules and RGD peptides as described in our previous work^32^. Briefly, 20 µL thiol-terminated PEG (PEG-SH) with a molar mass of 2000 g mol^−1^ (PEG 2k-SH) solution with concentration of 1 mM were mixed with 5 mL stable colloidal solution of CGNP clusters with OD 10 at 650 nm, corresponding to a input molar ratio of 400:1 between PEG 2k-SH molecules to GNPs in CGNP clusters. The mixed solution was kept undisturbed for 2 h at room temperature to enable sufficient conjugation of PEG 2k-SH molecules to the CGNP clusters via Au-sulfur bonds. After the reaction, partially PEGylated CGNP clusters were further conjugated with RGD peptides by adding to them 60 µL RGD peptide solution at a concentration of 1 mM, corresponding to an input molar ratio of 1200:1 between RGD peptides to GNPs in CGNP clusters. The mixed solution was kept at room temperature for further 2 h to ensure sufficient conjugation of RGD peptides onto unoccupied surface sites of the CGNP clusters. Then, the solution was centrifuged at 1000 × *g* for 30 min. Final optical density of the colloidal solution of CGNP clusters-RGD was adjusted to ~100 by resuspending the pellet with 4 mM borate buffer (pH 8.2) containing 5 mg/mL BSA after removing the supernatant. Furthermore, the amount of PEG 2k-SH and RGD peptide on CGNP clusters were determined using a method involving dynamic light scattering (DLS) size measurement as described in our previous paper^33^. It was concluded that the amount of PEG molecules on the surface of individual GNPs in CGNP clusters is about 400, and there were about 600–800 RGD peptides on surface of individual GNPs in CGNP clusters based on experimental data of hydrodynamic diameter changes of CGNP clusters as function of PEG amount (Supplementary Fig. S16) and RGD peptide amount (Supplementary Fig. S17).

### Physical and optical characterization of CGNP clusters-RGD

Hydrodynamic particle size distribution and surface zeta potential were evaluated with dynamic light scattering using a Zetasizer Nano ZS90 (Malvern Instruments, Malvern, Worcestershire, UK). TEM image was obtained using JEOL 2010F. The absorbance spectrum was determined from 400 to 1000 nm using a spectrophotometer (UV-3600, Shimadzu Corp., Japan). The infrared spectra of PEG 2k-SH, CGNP clusters, and CGNP clusters-RGD were analyzed using a PerkinElmer spectrum 100 FTIR spectrometer (PerkinElmer Inc., Waltham, MA) equipped with an attenuated total reflection (ATR) diamond. Scattering properties were determined using a custom-built system (see Supplementary Notes 3 and 4).

To evaluate the colloidal stability of the CGNP clusters-RGD, the absorbance of CGNP clusters-RGD was measured at different times up to 1 month. The stability of CGNP clusters-RGD in biological tissue was also evaluated (see Supplementary Note 5). To assess the photostability of the CGNP clusters-RGD, their absorbance spectra were measured after being illuminated with nanosecond pulsed laser of 650 nm at multiple pulse fluences of 0.005, 0.01, 0.02, and 0.04 mJ/cm^2^.

### Circulation time of CGNP clusters-RGD

The circulation kinetics of the synthesized CGNP clusters-RGD in blood were analyzed in rabbits in vivo. The rabbits were intravenously injected with CGNP clusters-RGD at concentrations of 2 and 4 mg/mL via the marginal ear vein. Post injection, 1 mL of blood samples were then collected at various time points ranging from 15 min to 72 h. The collected blood samples were dissolved in concentrated nitric acid and hydrochloric acid for analysis by inductively coupled plasma mass spectrometer (ICP-MS). The amount of CGNP clusters-RGD in diluted blood sample was measured using a Perkin-Elmer Nexion 2000 ICP-MS (Nexion 2000 s, Perkin-Elmer, MA, USA).

### Biodistribution and toxicity of CGNP clusters-RGD

To evaluate the toxicity of bare GNPs and CGNP clusters, nine groups of the animals (*N* = 3) were injected with 500 µL of bare conventional 20 nm GNPs and CGNP clusters-RGD at concentrations of 2.5, 5, 10, and 20 mg/kg (*N* = 24) via the tail vein. The weight of treated and untreated mice was recorded daily for 7 days. At day 7 post the injection, all of the animals were euthanized. Blood, heart, liver, lung, kidney, and spleen samples were harvested to evaluate the concentration of gold by ICP-MS (see Supplementary Notes 3–4) and mini chemistry panel analysis.

### Multimodal PAM and OCT instrumentation

A custom-built high-resolution multimodality PAM and OCT imaging system^34,35^ is developed and presented in the Supplementary Note 7, and Supplementary Fig. S1. In PAM mode, a tunable nanosecond pulsed laser ranging from 405 to 2600 nm (pulse repetition rate = 1 kHz and pulse duration = 3–5 ns) produced by a solid-state Q-switched Nd:YAG laser (NT-242, Ekspla, Lithuania) was utilized to illuminate the samples. The laser light was delivered through the optical system, filtered, and collimated to generate a homogeneous circular spot size of 2 mm. When the laser light shined into the eye, the laser beam was focused on the fundus with an estimated diameter of 20 µm. The average laser pulse fluence of approximately 0.01 mJ/cm^2^ at 578 and 650 nm was utilized to illuminate the eye, which is only half of the American National Standards Institute (ANSI) limit of the maximum permissible single laser pulse fluence on the retina (i.e., <=0.02 mJ/cm^2^ at 578 and 650 nm)^34,36^, leading to avoid undesirable effects such as thermoacoustic damage and thermal damage^37,38^. A 27 MHz in-house needle transducer (two-way bandwidth −60%; Optosonic Inc., Arcadia, CA, USA) was applied to detect the laser-induced PA signals^39^. By using the full-width at half-maximum (FWHM), the calibrated lateral and axial resolutions of the PAM system were estimated to be 4.1 um and 37.0 µm, respectively. An optical scanning galvanometer was used to scan the sample along the x- and y-directions. The PAM images were acquired at different wavelengths of 578 and 650 nm, which overlapped with the peak absorbance of hemoglobin and CGNP clusters-RGD. At each position, the laser-induced PA signal were amplified using a low-noise preamplifier (gain 57 dB, AU-1647, L3 Narda-MITEQ, NY) and digitized using a 200 MHz (PX1500-4, Signatec Inc., Newport Beach, CA). The obtained data was used to reconstructed PAM images in both 2D and 3D. 3D volumetric visualization was rendered using Amira software. Although the current instrument can achieve a single 3D volumetric PA image in less than 1 min, motion artifacts may occur during the data acquisition^30^. The acquisition speed can be increased by using a laser with higher repetition rates, which will be suitable for clinical translation.

High-resolution OCT images (both 2D and 3D) were also obtained using a spectral domain OCT equipment (Ganymede-II-HR, Thorlabs, Newton, NJ) with additional modification as described elsewhere^34,35^. In brief, a dispersion compensation glass and an ocular lens were added into the imaging platform (Supplementary Note 7 and Supplementary Figure S1). Dual super luminescent diodes (*λ* = 846 nm and *λ* = 932 nm) were used to illuminate into the samples. The calibrated lateral and axial resolutions were estimated to be 3.8 µm and 4.0 µm, respectively. Because of using two different light sources, the OCT light beam were coaxially aligned with PAM laser beam for guiding PAM and helping to interpret PAM results. The high-resolution B-scan OCT images can be obtained within 0.103 s with 512 × 1024 A-lines and the acquisition rate of 36 kHz.

### Demonstration of targeting choroidal neovascularization in vivo with CGNP clusters-RGD

All experimental protocols were approved by the Institutional Animal Care and Use Committee (IACUC) at the University of Michigan (PRO00008566). The animal experiments were employed in compliance with the guidelines of the Association for Research in Vision and Ophthalmology (ARVO) Statement on the care and use of laboratory animals in Ophthalmic and Vision Research. New Zealand White rabbits (male and female) with weighting of 2.0–3.2 kg and the age of 2–3 months were obtained from the Center for Advanced Models and Translational Sciences and Therapeutics (CAMTraST) at the University of Michigan Medical School. Two groups of rabbits (*N* = 12) were treated with either laser-induced retinal vein occlusion or subretinal release of human vascular endothelial growth factor (VEGF-165) (see Supplementary Note 6). At day 28 after treatment, CNV had developed^19^. Baseline PAM and OCT images were acquired pre- and post-intravenous (IV) injection of CGNP clusters-RGD and CGNP clusters without RGD (0.4 mL at mass concentration of 2.5 mg/mL) to visualize the morphology of retinal vessel networks. In PA imaging, nanosecond excitation laser with tunable wavelength output in 450–710 nm range was used. Prior to the injection, the CGNP clusters-RGD were sonicated for 5 min using an ultrasonic bath (Fisher Scientific, PA, USA) for de-agglomerating and dispersing CGNP clusters-RGD. The CGNP clusters-RGD suspension was then intravenously administrated into the rabbit in the marginal ear vein. PAM and OCT images were obtained at 2, 24, 48, 72 h and days 3, 4, 5, 7, 9, 11, and 14 post injection. The scanning areas were selected using the fundus camera, which minimized the variation between days. Each image was approximately a 4 × 4 mm field of view (step size = 15.5 µm). The acquisition time was about 65 s. At day 14 post injection, the rabbit blood was collected for liver function test and then the rabbit was sacrificed for histological and immunohistochemistry analysis. The PA image data acquired at different wavelengths was aligned and reconstructed in 3D using Amira software. The average PA signal within CNV was determined for each PA image.

### Immunostaining and electron microscopy analysis

To confirm the development of CNV, single immunohistochemistry slides were stained with three different antibodies: 1) a mouse monoclonal antibody raised against native human von Willebrand Factor (VWF) (VWF Mouse anti-Human, Clone: F8/86, Abnova Taipei, Taiwan); 2) glial fibrillary acidic protein (GFAP) (GFAP Mouse anti-Human, Pig, Clone: GA-5, Abnova, Taipei, Taiwan); 3) anti-alpha smooth muscle actin (α-SMA) antibody to stain smooth muscle cells in vessel walls (Abcam, Burlingame, CA, US). The slides were viewed under light microscope (DM6000, Leica Biosystems, Nussloch, Germany). Images were digitally captured using Leica Application Suite software (LAS X, Leica Biosystems, Nussloch, Germany) and displayed in Supplementary Fig. S18. For electron microscopy analysis, liver specimens were fixed in 2.5% glutaraldehyde solution in a 0.1 M Sorensen’s phosphate buffer (pH = 7.4) and washed three times with Soren’s buffer solution at concentration of 0.1 M. Then, the samples were dehydrated in acetone and polymerized for electron microscopy analysis. Samples were cut into ultrathin (~70 nm) slices using ultra-microtome (Sorvall MT-2B, NY, USA), mounted onto 200 mesh fine bar hex grids without the base layers, and were stained with uranyl acetate and lead citrate. Transmission electron microscope (JOL-JEM 1400 Plus, Japan Electron Optic, Tokyo, Japan) was utilized to acquire the electron micrographs.

### Statistical methods

The hypothesis that the administration of CGNP clusters-RGD can target CNC and increase both PA and OCT signal over time compared with the control group without injection of nanoparticles was evaluated using a random-effects regression model. The experiments were implemented and repeated three times. The Student’s t-test at each condition was employed to determine any significant difference image contrast pre- and post injection. The final data points were represented as the mean ± standard deviation (SD). *P-*values of <0.05 indicated as statistically significant.

## Supplementary information

Supplementary Information

Description of Additional Supplementary Files

Supplementary Movie 1

## Source data

Source Data
