# Supplementary material for: Chain-like gold nanoparticle clusters for multimodal photoacoustic microscopy and optical coherence tomography enhanced molecular imaging
Source: Nat Commun. 2021 Jan 4;12:34. doi: 10.1038/s41467-020-20276-z (PMC7782787; doi:10.1038/s41467-020-20276-z)
Supplement: Supplementary file 1 — Supplementary Information [file 41467_2020_20276_MOESM1_ESM.doc]

**Supplementary Information for Notes, Figures, and Movie**

**Chain-like Gold Nanoparticle Clusters for Multimodal Photoacoustic Microscopy and Optical Coherence Tomography Enhanced Molecular Imaging**

**Van Phuc Nguyen1,4, Wei Qian2, Yanxiu Li1, Bing Liu2, Michael Aaberg1, Jessica Henry1, Wei Zhang3, Xueding Wang3*, and Yannis M. Paulus1,3* ­­­**

1Department of Ophthalmology and Visual Sciences, University of Michigan, Ann Arbor, MI 48105, USA

2IMRA America Inc, 1044 Woodridge Ave., Ann Arbor, MI 48105, USA

3Department of Biomedical Engineering, University of Michigan, Ann Arbor, MI 48105, USA

4NTT-Hitech Institutes, Nguyen Tat Thanh University, Ho Chi Minh City, Vietnam

*Corresponding Authors:

Yannis M. Paulus, M.D., F.A.C.S.

Department of Ophthalmology and Visual Sciences

Department of Biomedical Engineering

University of Michigan

1000 Wall Street

Ann Arbor, MI 48105, USA

Email Address: ypaulus@med.umich.edu

Xueding Wang, Ph.D.

Department of Biomedical Engineering

Department of Radiology

University of Michigan

Email Address: xdwang@umich.edu

**Supplementary Figures**


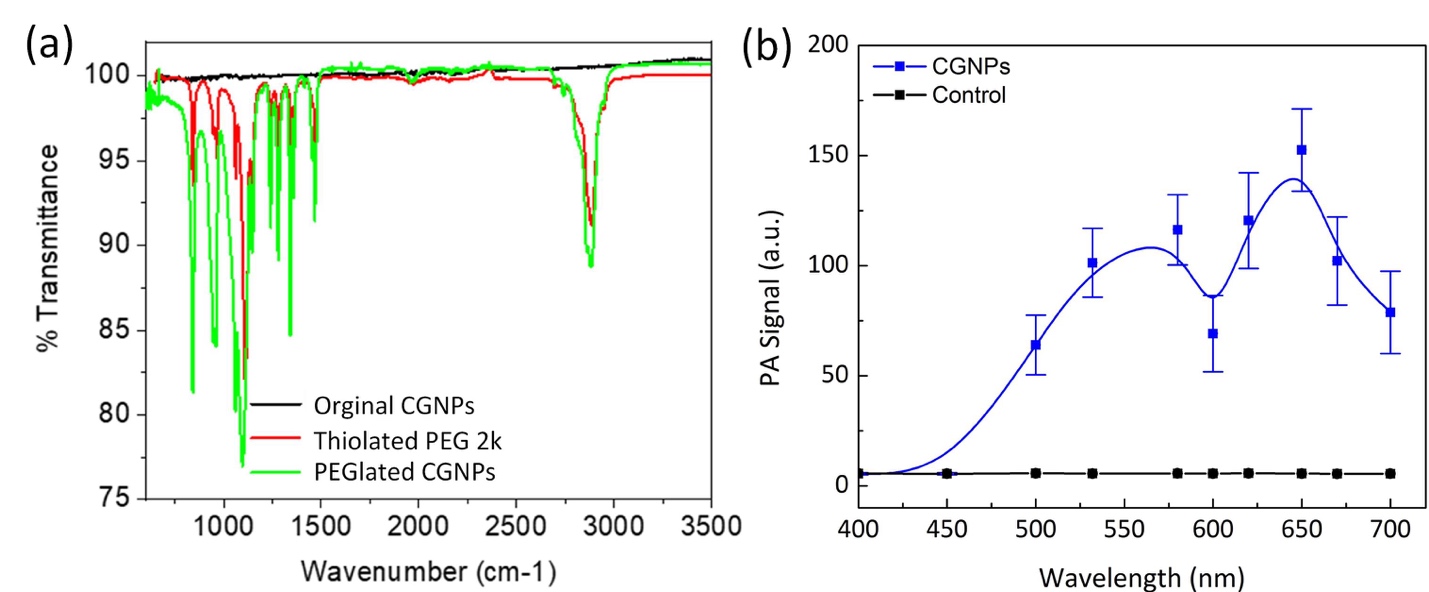


**Supplementary Figure S1.** (a) Fourier transformed infrared spectroscopy (FTIR) of PEGylated chain-like gold nanoparticle (CGNP) clusters. (b) Graph of PA signal as a function of excitation wavelength ranging from 400 to 700 nm. Error bar represents the standard deviation measured from 20 independent regions of interest on PAM images. Source data are provided as a Source Data file.

*
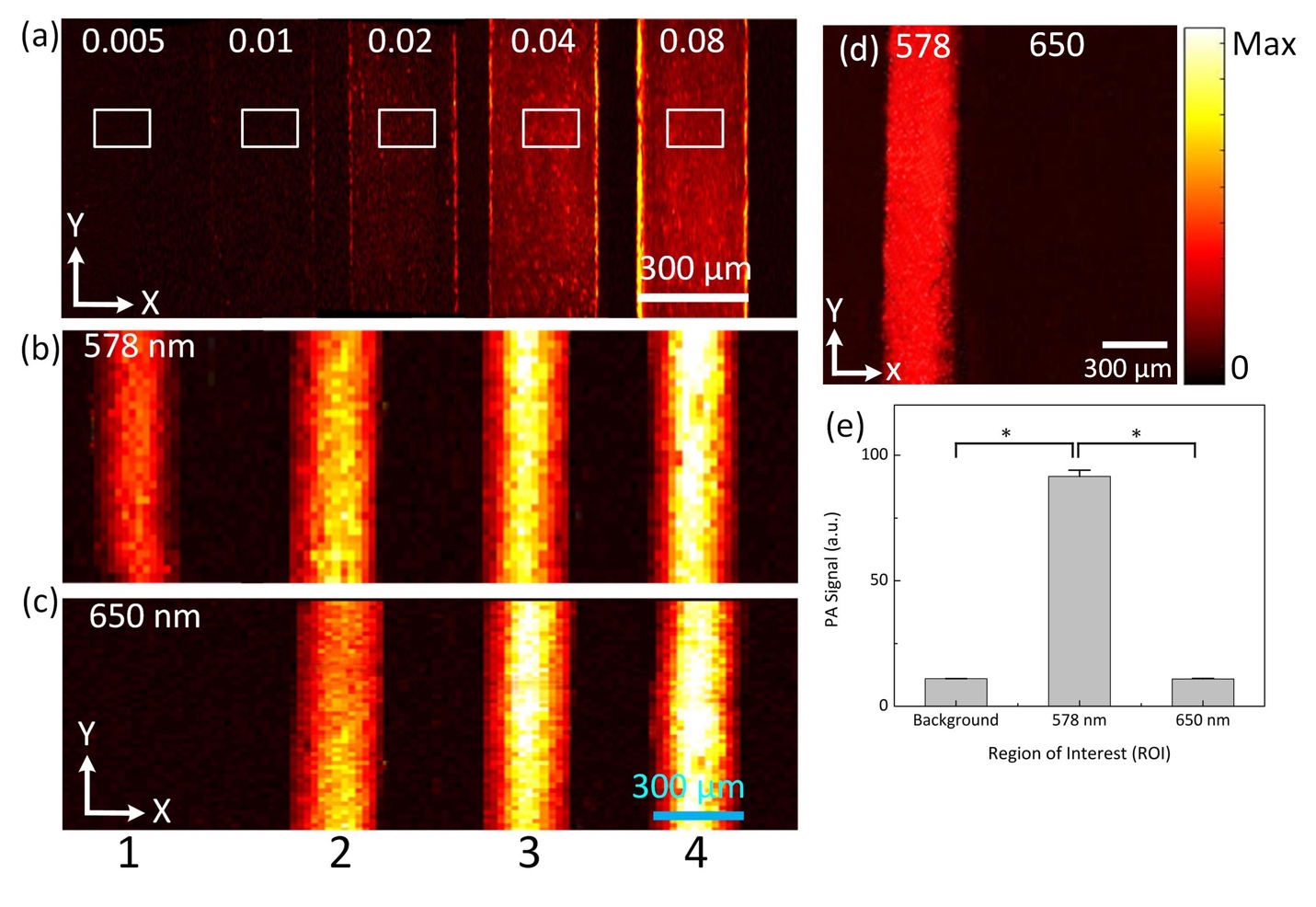
*

**Supplementary Figure S2.** In vitro quantitative analysis of photoacoustic response of CGNP clusters: (a) PA image of phantom made from silicone tubes filled with CGNP clusters at different concentrations ranging from 0.005 to 0.08 mg/mL obtained at a wavelength of 650 nm with laser fluence of 0.01 mJ/cm2. White rectangles show the region of interest (ROI) used to determine the PAM amplitude. PA images of blood filled phantom containing CGNP clusters with different final concentrations of 0 mg/mL (sample 1), 0.02 (sample 2), 0.04 (sample 3), and 0.08 mg/mL (sample 4) acquired at 578 nm (b) and 650 nm (c). The PA images acquired at 578 nm from the blood-filled phantom containing CGNP clusters showed higher contrast compared with that of phantom filled with blood only. In addition, the phantom filled with blood only were undetectable at the wavelength of 650 nm whereas the blood-filled phantom containing CGNP clusters at low final concentration of 0.02 mg/mL were clearly visualized with high contrast. (d) PAM image of phantom sample poured with fresh rabbit blood obtained at 578 and 650 nm. The sample acquired at 578 shows high contrast whereas no signal was observed on the PAM image acquired at 650 nm due to low absorption of hemoglobin at 650 nm. (e) Graph of PA signal amplitudes measured from 3 different ROIs on the PAM image: background, blood sample at 578 and 650 nm, respectively. Error bar represents the standard deviation measured from five independent samples (p<0.001). Source data are provided as a Source Data file.

*
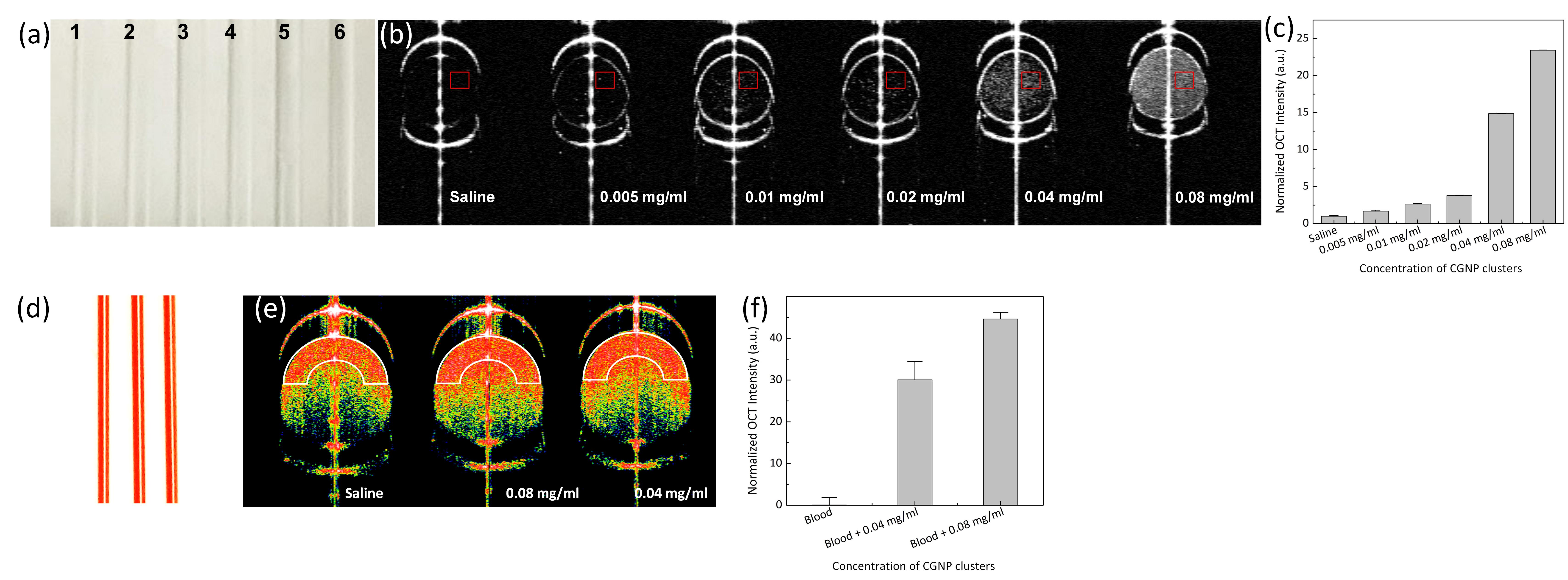
*

**Supplementary Figure S3.** *In vitro* quantitative analysis of OCT response of CGNP clusters: (a) photograph of phantom filled with CGNP clusters at different concentrations ranging from 0 (saline) to 0.08 mg/mL with an increase interval of 0.005 mg/mL. (b) corresponding B-scan OCT image. Red rectangles show the region of interest (ROI) used to determine OCT intensity. (c) plot of the growth of OCT signal acquired from the red ROI shown in Figure b as increasing the concentration of CGNP clusters. (d) photograph of phantoms filled with fresh rabbit blood and mixture of rabbit blood and CGNP clusters at various concentrations. (e) cross-sectional OCT images acquired along the dotted line in Figure d. (f) quantitative analysis of OCT signal at various ROI on the acquired B-scan OCT images shown in Figure e (i.e., blood only and mixtures of blood and CGNP clusters). Source data are provided as a Source Data file.


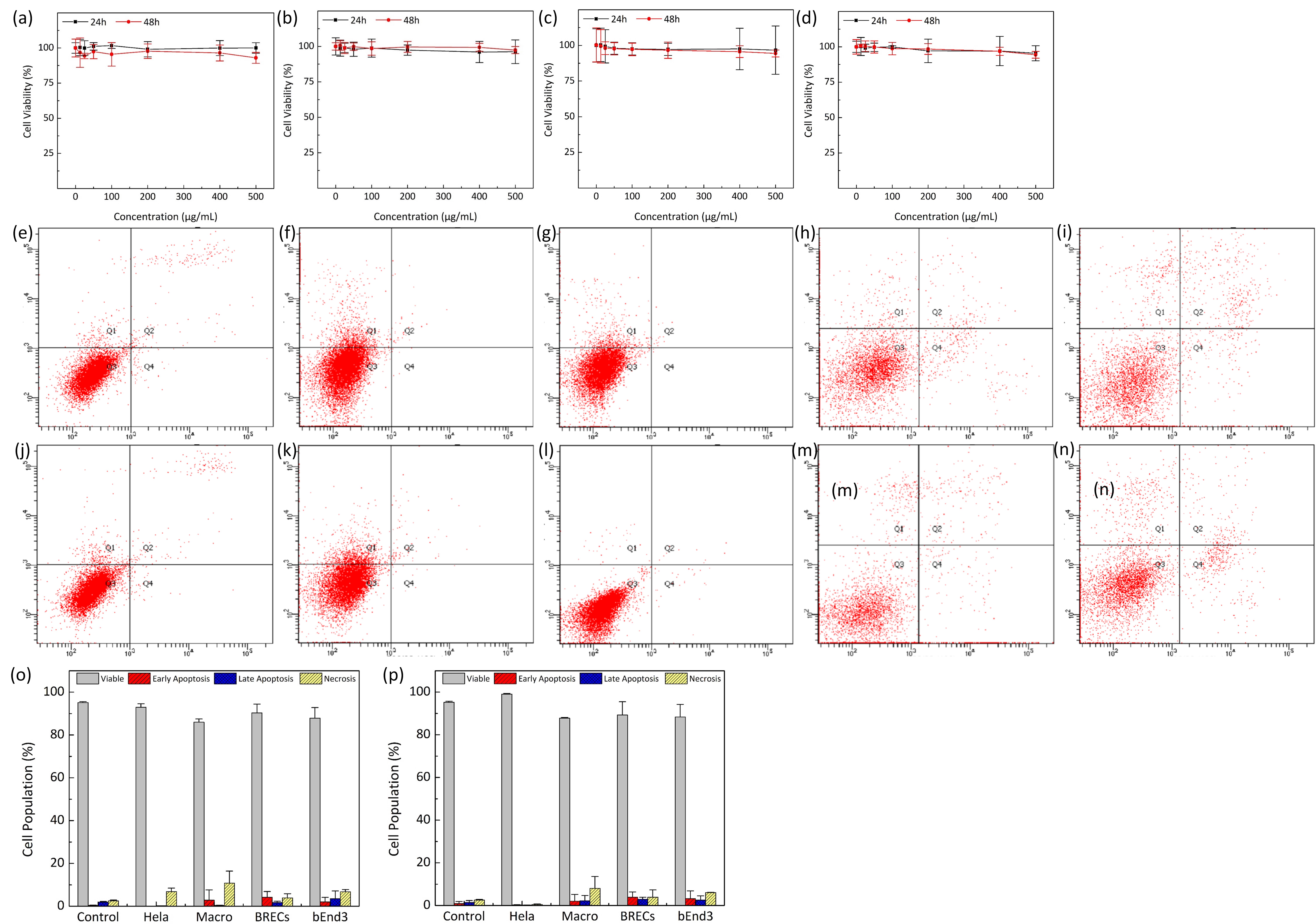


**Supplementary Figure S4.** Cytotoxicity and Biocompatibility of CGNP clusters-RGD: (a-d) cell viability assays of macrophages (Raw. 467), HeLa cells, bovine brain endothelial (b.End3), and bovine retinal endothelial (BRECs) cells after treatment with CGNP clusters-RGD at various concentrations (0 (control), 12.5, 25, 50, 100, 200, 300, 400, and 500 µg/mL) for two incubation periods, 24 h and 48 h. (e-j) Flow cytometry analysis on apoptosis and necrosis of Raw. 467, HeLa, b.End3, and BRECs cells after treatment with CGNP clusters-RGD at concentration of 400 µg/mL for 24h and 48h (j-n). Note that (e) and (j) are control group without treatment with CGNP clusters-RGD. (o) and (p) quantitative analysis of percentage of gated cells for viable, necrotic, early apoptosis and late apoptosis (p < 0.05) after treatment for 24 h and 48 h, respectively. Source data are provided as a Source Data file.

*
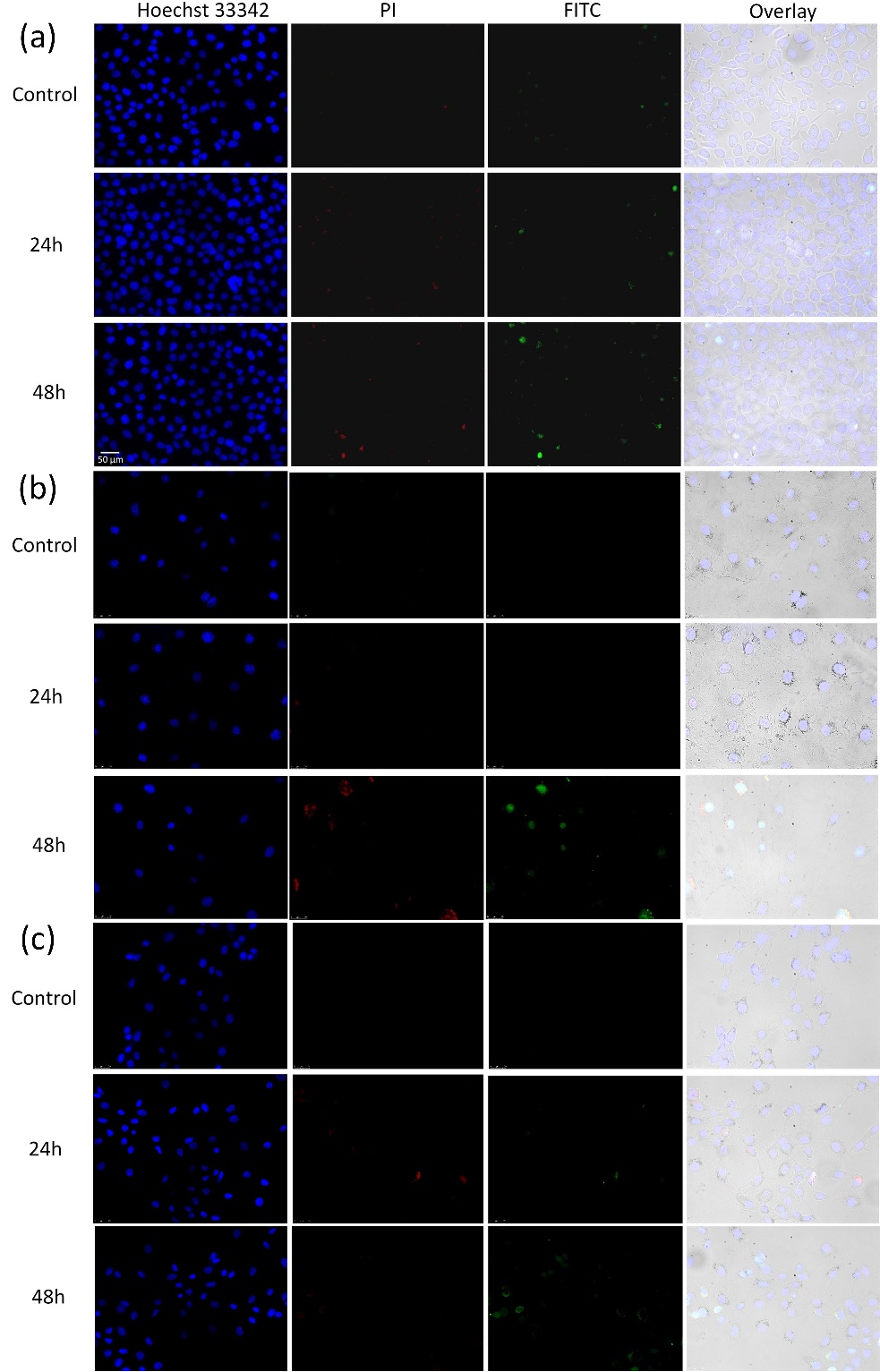
*

**Supplementary Figure S5.** Cytotoxicity studies of CGNP clusters-RGD: effect of CGNP clusters-RGD on HeLa cells (Figure a), brain endothelial cells (bEnd.3) (Figure b), and bovine retinal endothelial cells (BRECs) (Figure c) at various incubation times of 24 and 48 h, respectively. Confocal fluorescence microscopy images of the cells stained with Hoechst (33342), propidium iodide (PI), and Annexin-V FITC after treatment with CGNP clusters-RGD at concentration of 50 µg/mL, respectively. The right images are the overlaid fluorescence and bright-field images. Confocal fluorescence analysis used to detect the state of cell population such as live, apoptotic and dead cells after incubation with CGNP clusters-RGD. The fluorescence color represents the nucleic morphology of cell affected by CGNP clusters-RGD. Blue fluorescent color shows the morphology of cell’s nuclei stained with Hoechst 33342. The dead cells were stained with PI and displayed red color. Apoptotic cells were stained with FITC and displayed green color on the image. The fluorescence images were obtained at emission wavelengths of 461 nm for Hoechst 33342, 530 for FITC, and 617 nm for PI, under laser excitation at 350 nm for Hoechst 33342, 470 for FITC and 535 nm for PI, respectively.


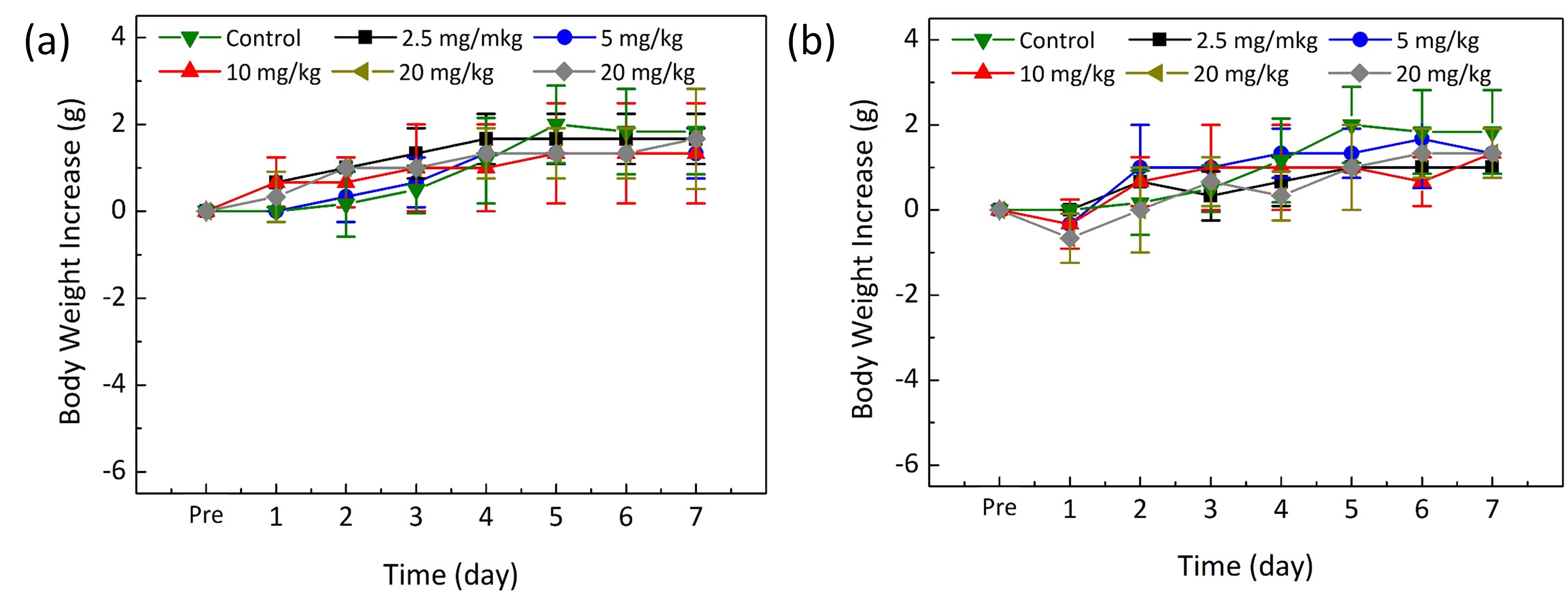


**Supplementary Figure S6.** Body weight as a function of time after treatments with various conditions: (a) mice untreated without (control) and with PEG-GNPs at 2.5, 5, 10, 20, and 40 mg/kg, (b) mice groups treated without and with CGNP clusters-RGD at 2.5, 5, 10, 20, and 40 mg/kg. The body weight was measured every day over a period of 7 days after each treatment. Data shown as mean ± SD (N=3, p < 0.01). Source data are provided as a Source Data file.


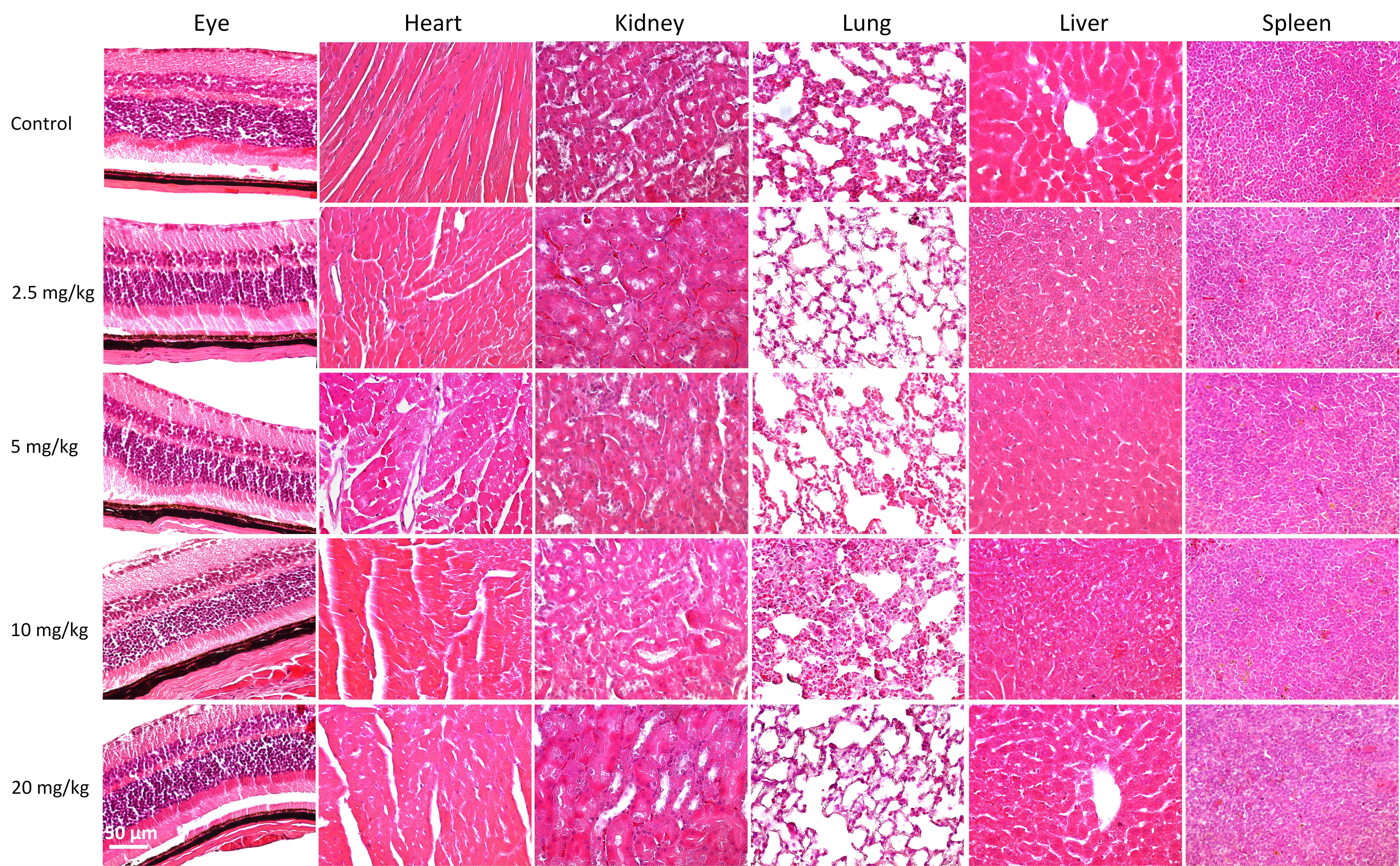


**Supplementary Figure S7.** H&E image of various mice tissues obtained from 5 different groups: control (c), and treated with 2.5, 5, 10, and 20 mg/kg of CGNP clusters-RGD.

*
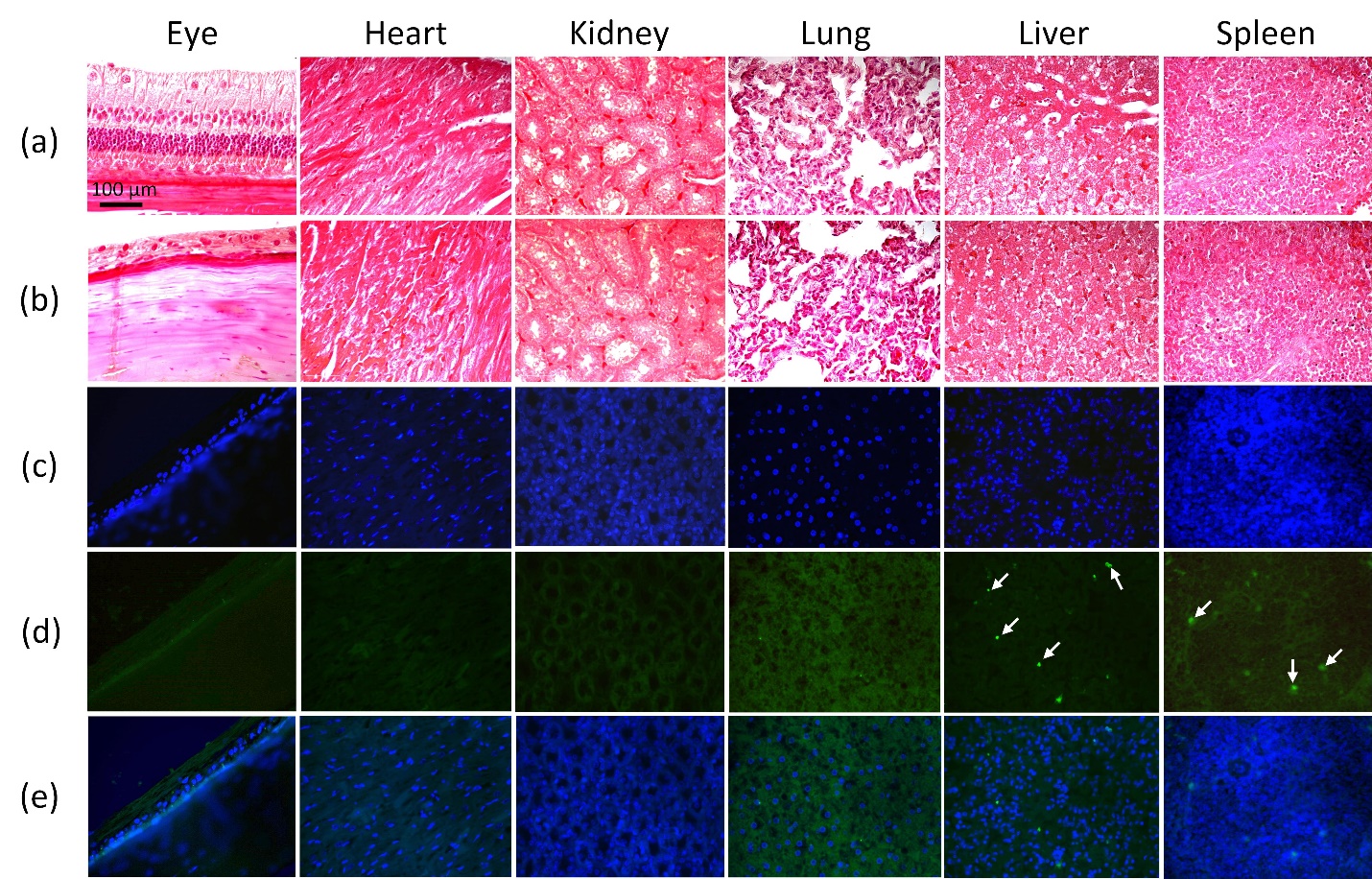
*

**Supplementary Figure S8.** Histological TUNEL assay and H&E staining images of different rabbit tissues of control group (a) and treated group with CGNP clusters-RGD (b). (c-d) TUNEL assay of the treated group. Blue fluorescent color shows the morphology of cell nuclei stained with DAPI (c). Green fluorescent indicates the position of apoptotic cells stained with FITC marked as white arrows (d). (e) overlay images.


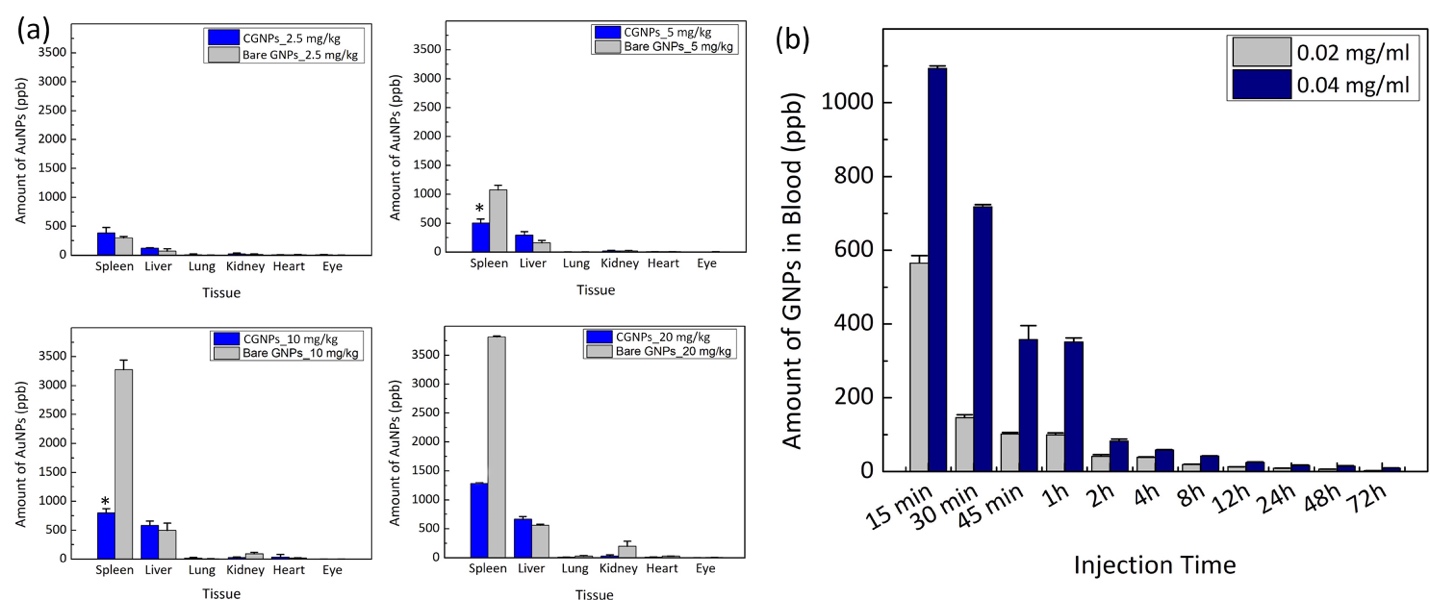


**Supplementary Figure S9**. (a) quantitative analysis of amount of GNPs -RGD and CGNP clusters-RGD accumulated in tissues by using wet digestion protocol ICP-MS method[1](#_ENREF_1). Data expressed as mean ± SEM (N=3). (b) *in vivo* circulation time of CGNP clusters-RGD in rabbit blood after injection of CGNP clusters-RGD at concentration of 2 and 4 mg/mL. Source data are provided as a Source Data file.

*
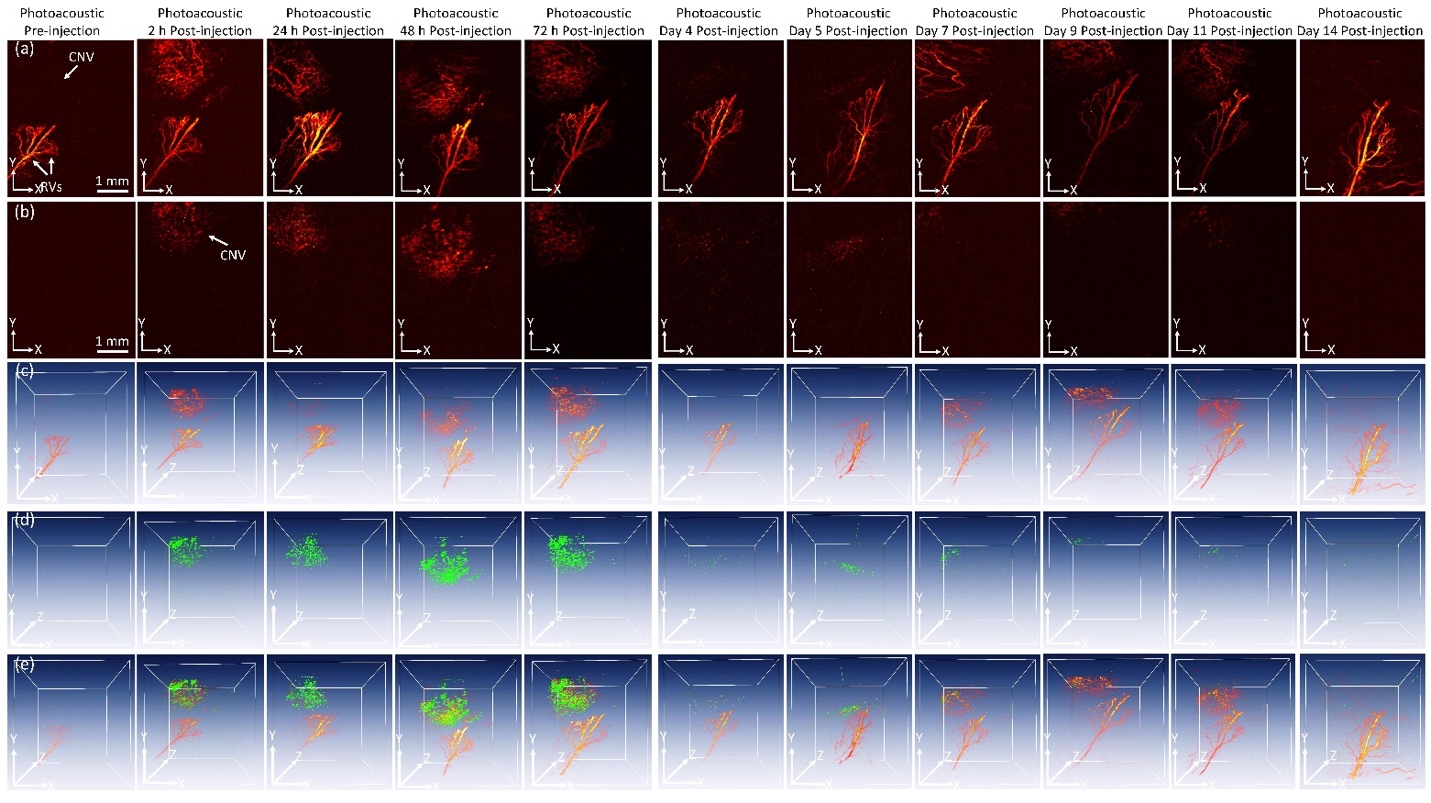
*

**Supplementary Figure S10.** 3D reconstruction PAM images before and after the injection of CGNP clusters-RGD at different time points at 2 h, 24 h, 48 h, 72 h, Day 4, 5, 7, 9, 11 and 14. (a) PAM images obtained with excitation laser wavelength at 578 nm and pulse fluence of 0.01 mJ/cm2. Both retinal vessels (RVs) and choroidal neovascularization (CNV) were clearly visualized with high image contrast. (b) PAM images acquired at 650 nm. CNV was clearly detected whereas RVs were invisible on PAM image. (c) and (d) 3D rendering PAM images. (e) Overlay PAM images acquired at 578 and 650 nm. Pseudo-green color indicates the distribution of CGNP clusters-RGD.


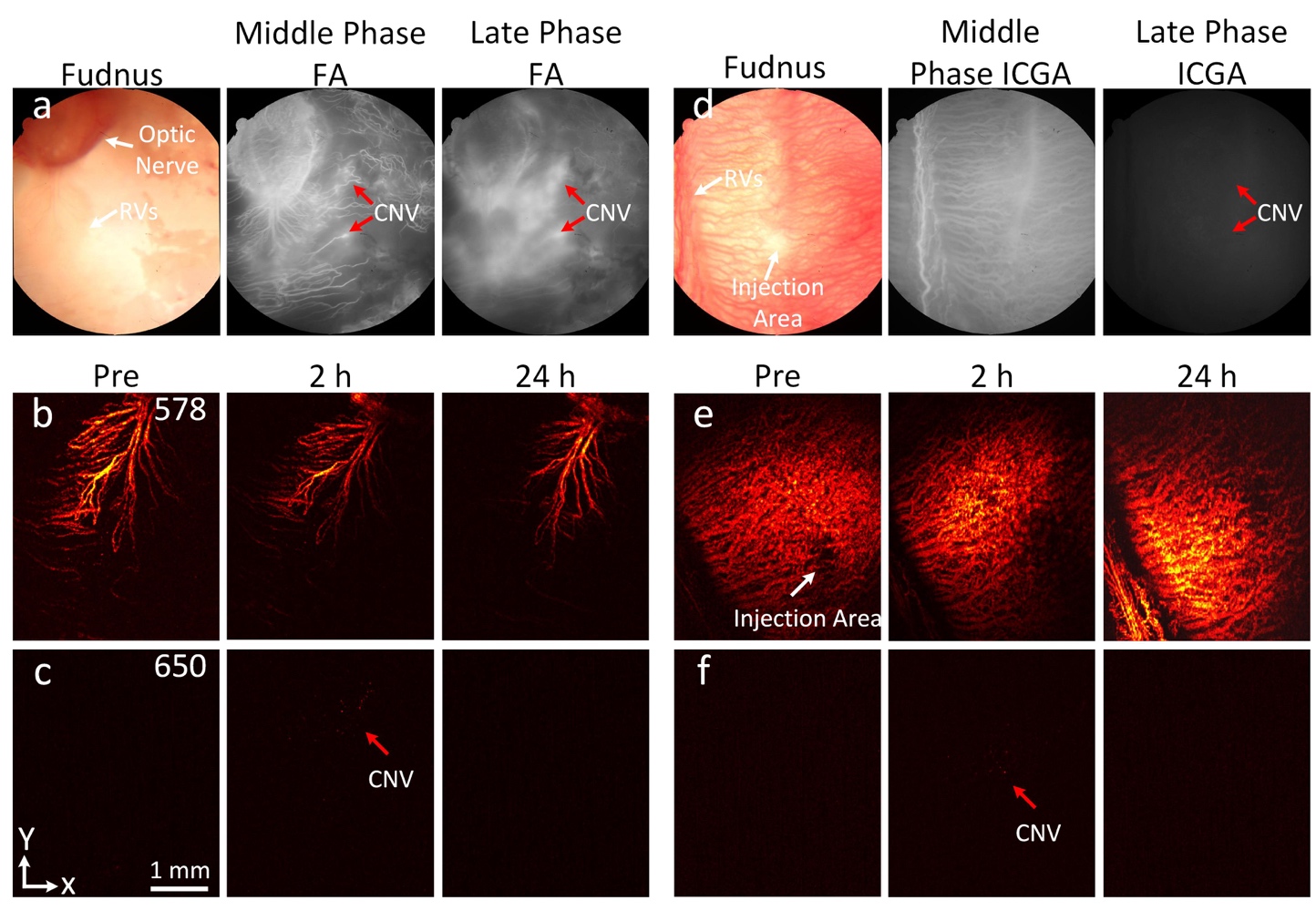


**Supplementary Figure S11.** *In vivo* PAM imaging of negative control group before and after injection of CGNP clusters without conjugated with RGD ligands (N=6). (a) Color fundus photography (left) of rabbit with retinal vein occlusion (RVO model, middle phase fluorescein angiography (FA) and late phase FA (right). Fundus color shows major retinal vessels and optic nerve (white arrow). FA images show the position of new developed choroidal neovascularization (CNV) as depicted by red arrows. (b–c) PAM images of CNV acquired at the excitation wavelength of 578 and 650 nm pre- and post-injection of CGNPs clusters (0.4 mL, 5 mg/mL) at 2 h and 24 h (N=3). Red arrow shows the detected CNV. The CNV were not obviously visualized on the PAM obtained at 650 nm after the injection of nanoparticles due to lack of targeting peptides. The detected signal occurred at 2 h post-injection may come from the extravasation of CGNPs at CNV as a result of the EPR effect. (d) Color fundus photograph of rabbit eye after subretinal injection of VEGF at day 7 before the injection of CGNPs cluster (left), middle and late phase indocyanine green angiography (right). White circle represents the injection region. Red arrows indicate the developed CNV. (e–f) PAM images acquired at 578 and 650 nm at different times points: pre, 2 h and 24 h post-injection of CGNPs clusters (0.4 mL, 5 mg/mL) (N=3).


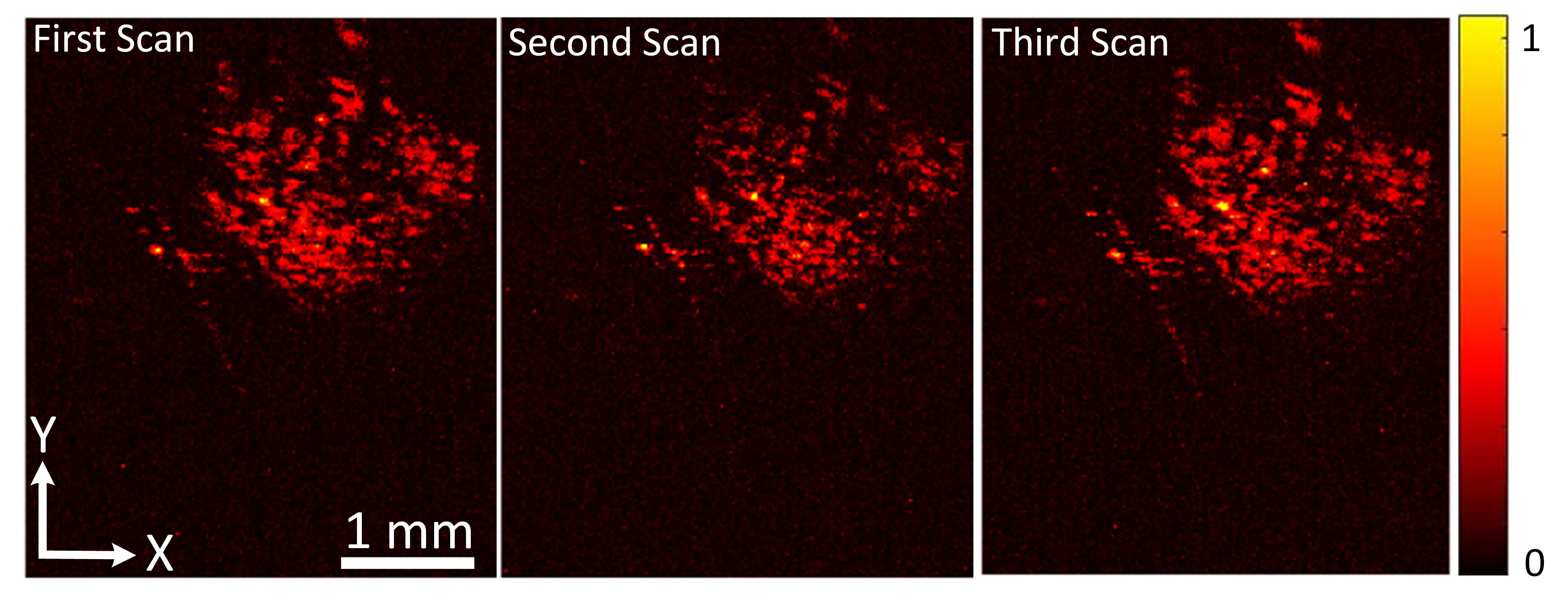


**Supplementary Figure S12.** *In vivo* photostability. ROI was scanned for 3 times under nanosecond pulsed laser illumination with the same fluence. The scanning time acquisition is approximately 1 min to achieve each volumetric image. Thus, the total scanning time is about 3 min.


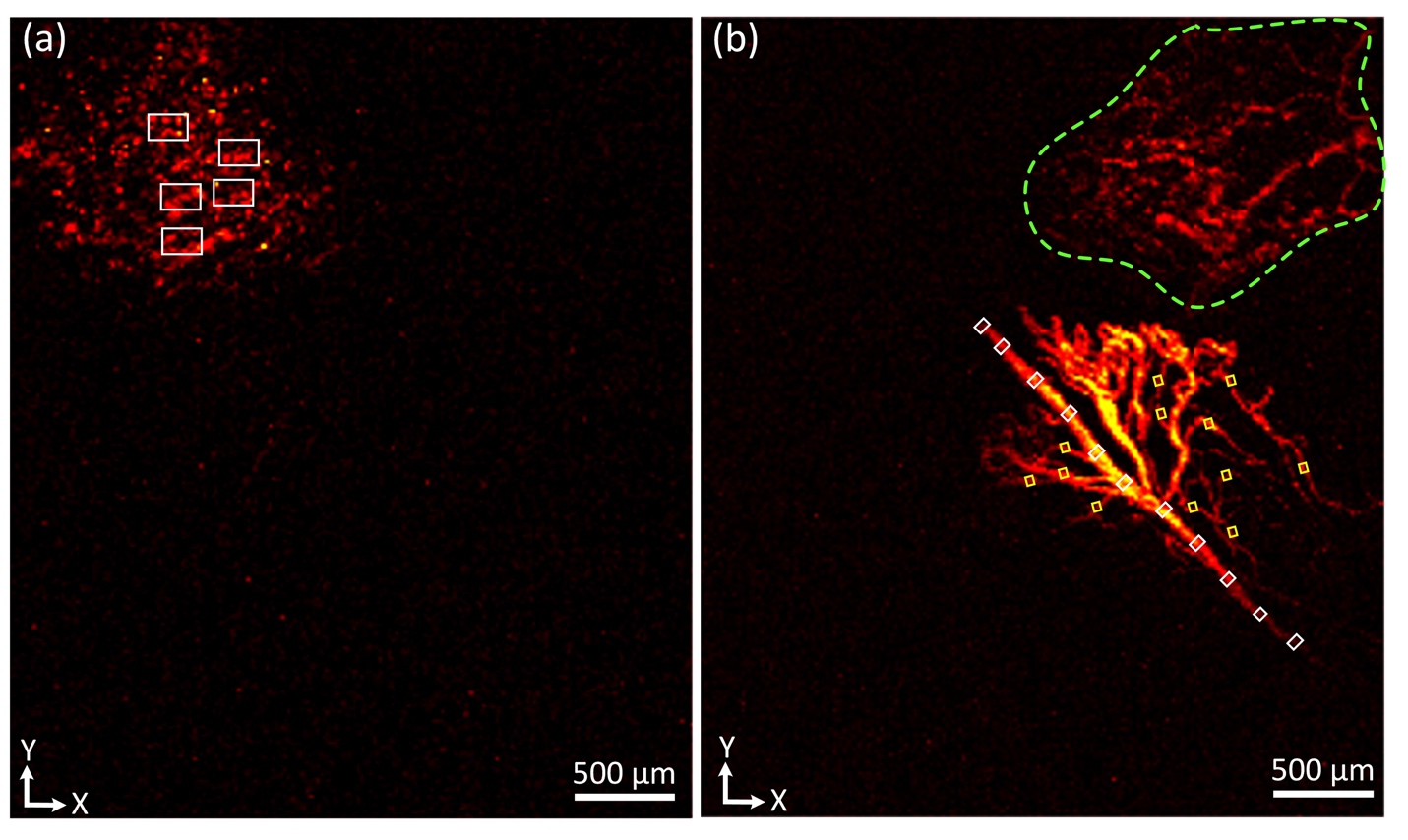


**Supplementary Figure S13.** Region of interest (ROI) to determine average PAM signal, vessels diameter and CNV area. (a) Five different ROI was isolated at the position of CNV (white rectangle). (b) Dozens of ROI were randomly selected along the retinal vessels (white rectangles) and capillaries (yellow rectangles) in PAM images. Green dotted line shows the selected ROI to determine the density of CNV.


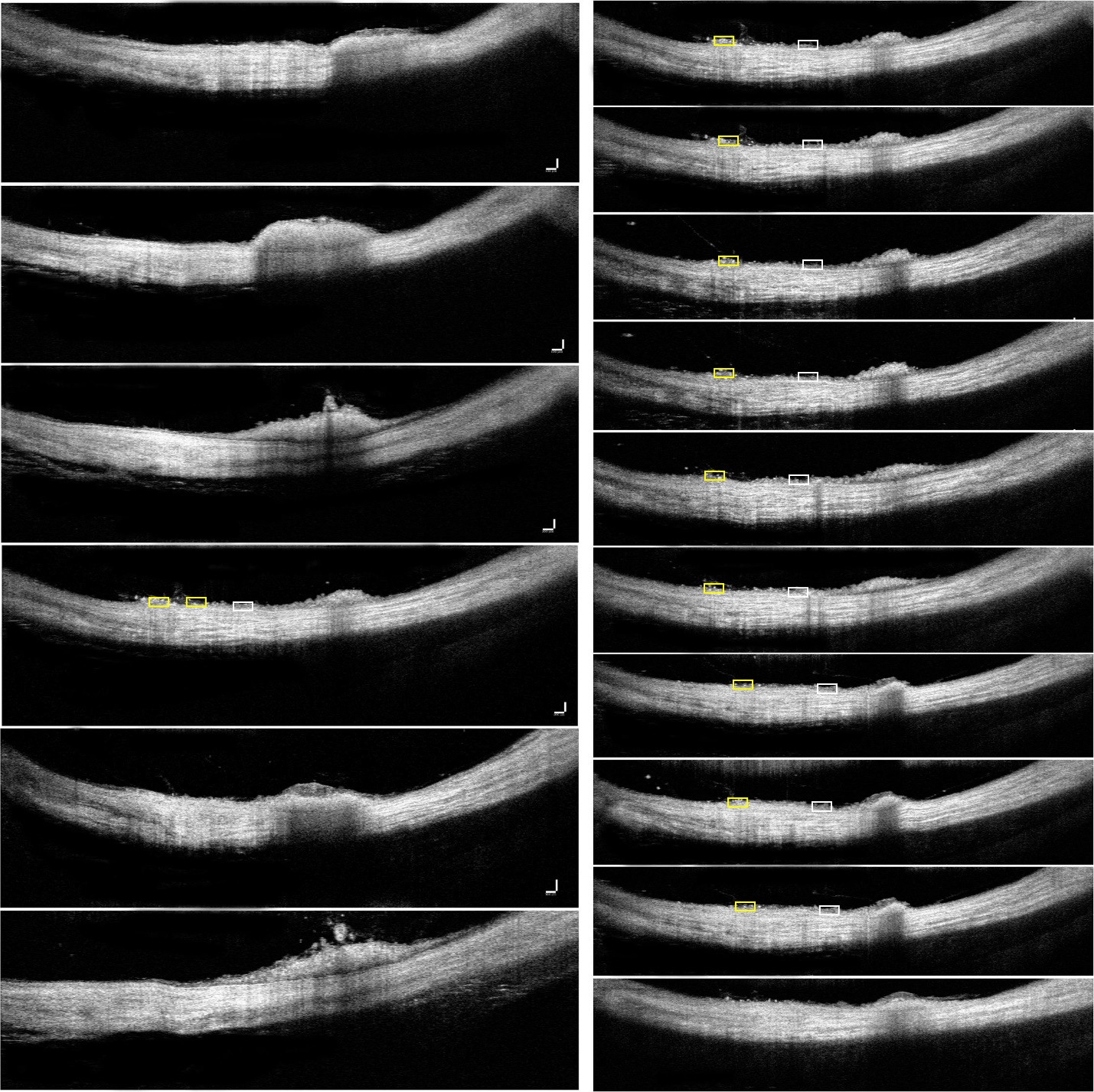


**Supplementary Figure S14.** *In vivo* OCT signal measurement: regions of interest were selected to determine the intensities of OCT before and after administration of CGNP clusters-RGD. Yellow rectangles show the ROI of CGNP clusters-RGD whereas white rectangles show the ROI of native tissues.


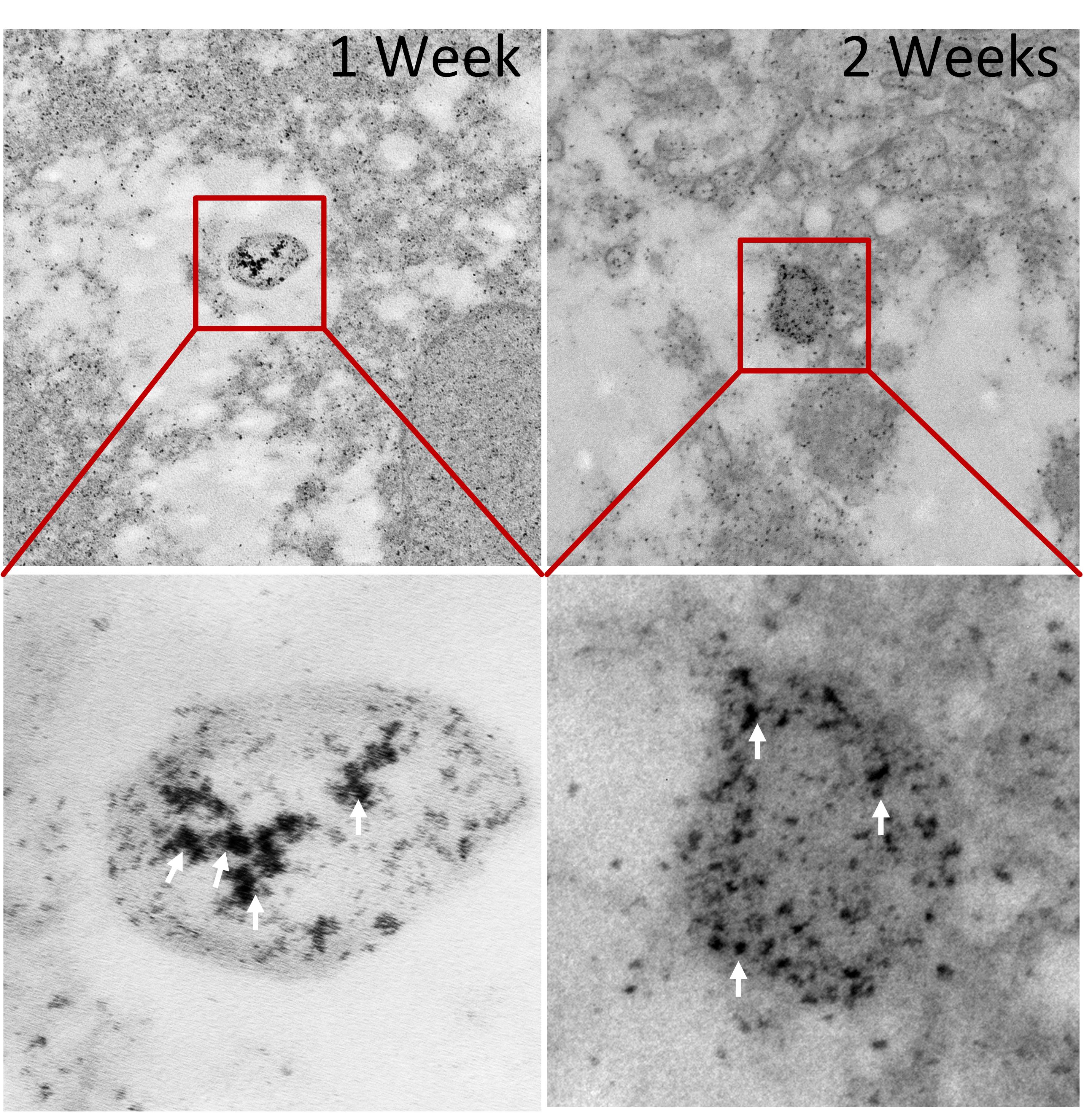


**Supplementary Figure S15.** Transmission electron microscopy (TEM) images of liver tissues. White arrows indicate the location of nanoparticles.


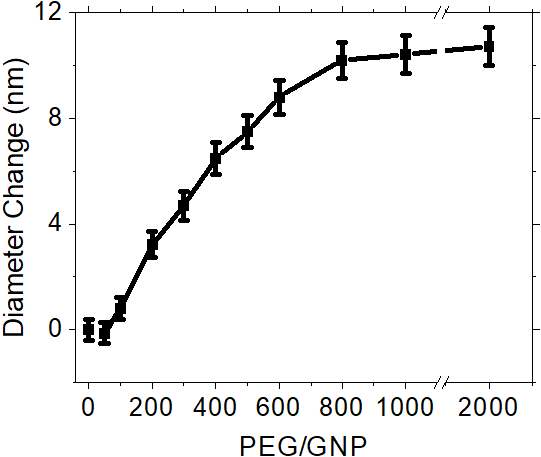


**Supplementary Figure S16**. Diameter change of the CGNP clusters after being PEGylated with different amount of PEG measured by DLS. PEG/GNP represents the molar ratio of PEG to individual GNPs in the CGNP clusters and PEG/GNP = 400 was used throughout the experiment to keep the stability of the CGNP clusters and leave enough surface space for subsequent conjugation of RGD peptides.


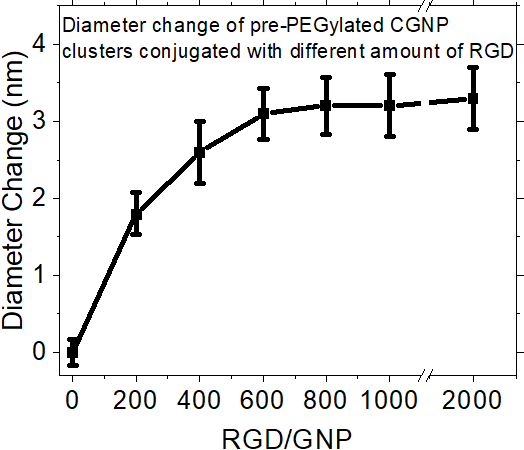


**Supplementary Figure S17**. Diameter change of pre-PEGylated CGNP clusters after further conjugation with different amount of RGD peptide measured by DLS (a molar ratio of PEG/GNP = 400 was used to produce pre-PEGylated CGNP clusters).


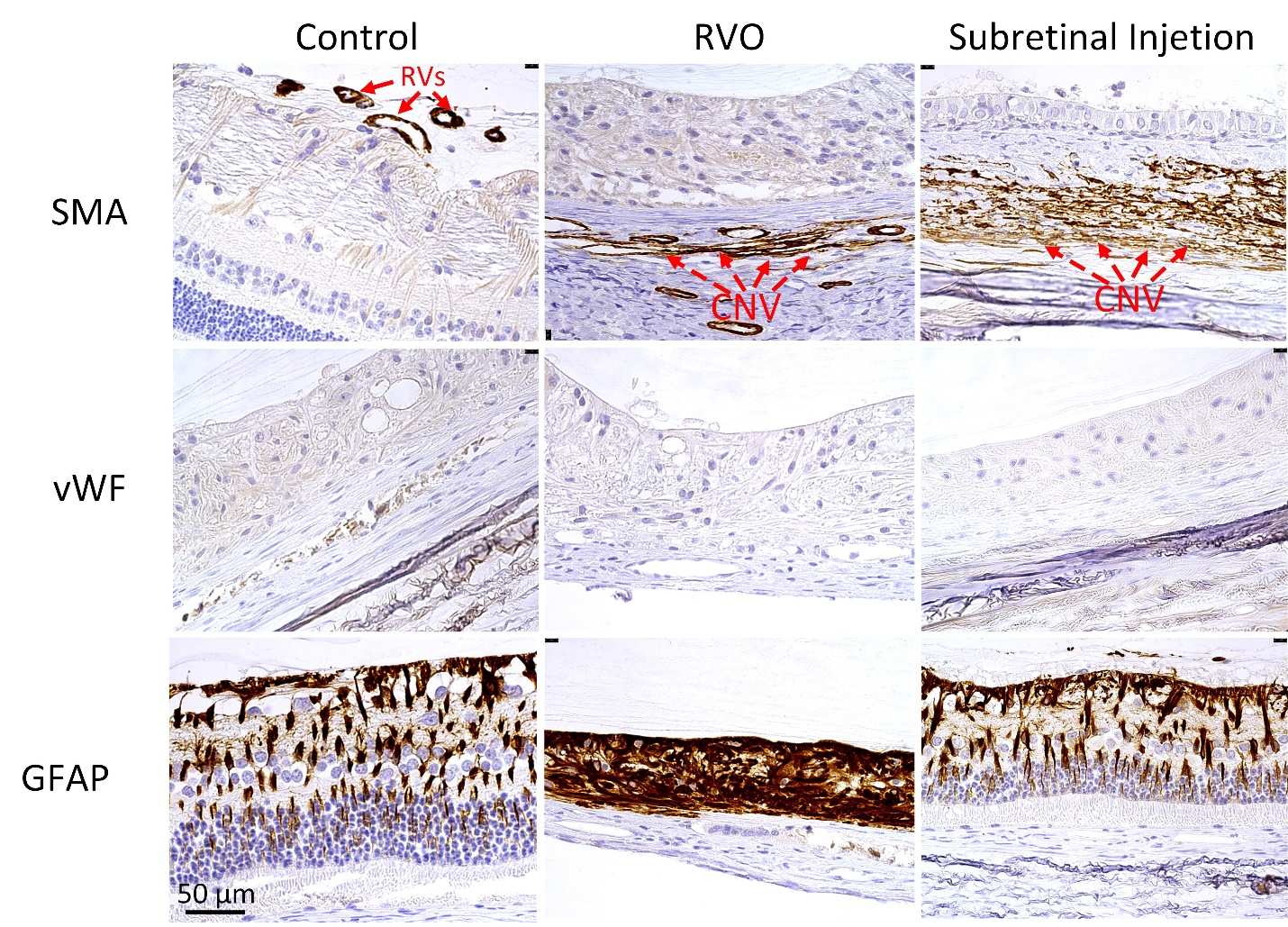


**Supplementary Figure S18.** Immunohistochemistry analysis. Immunohistochemically staining for SMA showed the presence of both abnormal dilated blood vessels (white dotted arrows) and new developed CNV

**Supplementary Movie:**

**Supplementary Movie 1:** 3D image reconstruction of retinal and choroidal neovascularization at day 3 after administration of CGNP clusters-RGD at final concentration of 0.02 mg/mL in rabbits

**Supplementary Note 1: Chemical materials and Instrumentations**

Rose Bengal was obtained from Sigma-Aldrich (Sigma, St. Louis, Mo, USA). 10% fluorescein sodium and indocyanine green (ICG) were purchased from Akorn (Akorn, Lake Forest, IL, USA). Ketamine was ordered from the University of Michigan Pharmacy from JHP Pharmaceuticals (JHP Pharmaceuticals, Rochester, MI, USA). Xylazine was acquired from MWI Animal Health (Anased®Boise, ID, USA). Thiol-terminated PEG (PEG-SH) with a molar mass of 2000 g mol−1 (PEG 2k-SH) was purchased from Creative PEGWorks (Chapel Hill, NC, catalog # PLS-605). PEG 2k-SH was in powder form and dissolved in deionized (DI) water prepared using Milli-Q Academic water purification system (Billerica, MA) having an electric conductivity less than 0.7 μS cm−1. Both cysteine-modified (RGD)4 peptides with an amino acid sequence RGDRGDRGDRGDPGC and pentapeptide ligand with an amino acid sequence CALNN having purity higher than 95% were custom-synthesized by RS synthesis LLC (Louisville, KY). Cysteamine (CAS Number: 60-23-1) with purity higher than 95% was purchased from Sigma-Aldrich. PEG, peptides, and cysteamine were in powder form and dissolved in deionized water having an electric conductivity less than 0.7 μS cm−1. All solutions were freshly made as needed and used within twelve hours. The bulk gold target (16 mm long, 8 mm wide, 0.5 mm thick, and 99.99% purity) used in the laser ablation experiment was purchased from Alfa Aesar (Ward Hill, MA). EndoGRO were ordered from Vec Technologies (Rensselaer, NY, USA). Complete MCDB-131, trypsin–ethylenediaminetetraacetic acid (trypsin-EDTA), antibiotics/antimycotics, fetal bovine serum (FBS), phosphate-buffered saline (PBS) were purchased from Gibco BRL, Life Technologies (Grand Island, NY, USA). Heparin, epidermal growth factor (EGF), fibronection, tylosin, sodium bicarbonate, 3-(4,5-Dimethyl-2-thiazolyl)-2,5-diphenyl-2H-tetrazolium bromide (MTT), dimethyl sulfoxide (DMSO), Hoechst 33342, and propidium iodide (PI), Annexin-V FITC, Dulbecco’s modified eagle’s medium (DMEM) were obtained from Sigma-Aldrich (Sigma, St. Louis, Mo, USA). Balanced salt solution (BSS) was purchased from Fisher scientific (Fishersci Inc., NH, USA). All chemicals were used as received without further purification.

Images of the colloidal GNPs were recorded using a TEM (JEOL 2010F, Japan) at an accelerating voltage of 100 kV. Nanoparticle hydrodynamic diameter size and zeta potential were characterized via DLS analyses using a Nano-ZS90 Zetasizer (Malvern Instrument, Westborough, MA). UV–vis absorption spectra were recorded by a spectrophotometer (UV-3600, Shimadzu Corp., Japan). The infrared spectra were measured using a PerkinElmer spectrum 100 Fourier transformed infrared spectroscopy (FTIR) spectrometer (PerkinElmer Inc., Waltham, MA) equipped with an attenuated total reflection (ATR) diamond.


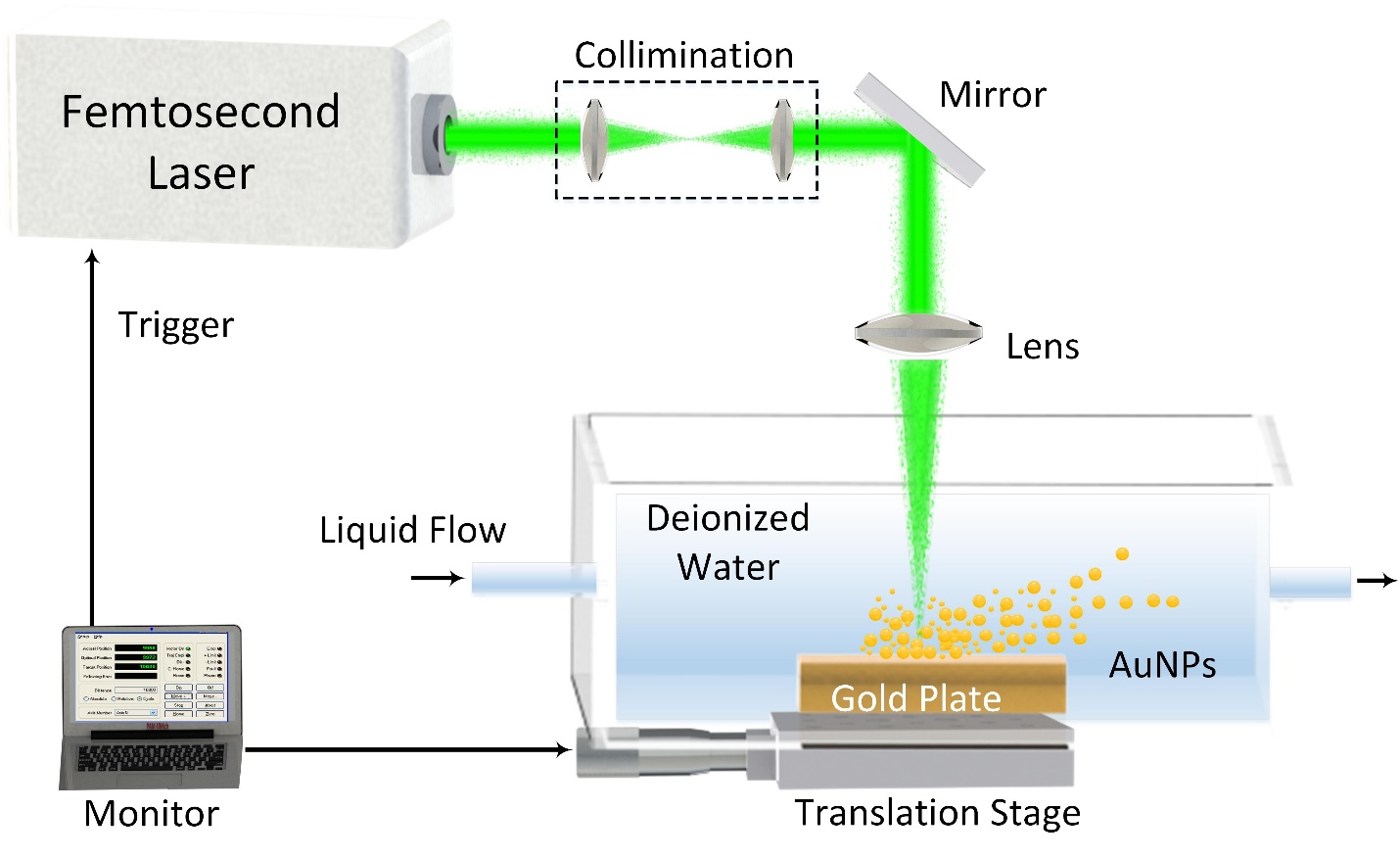


Fig S19. Ultra-pure colloidal gold nanoparticles fabrication. Colloidal gold nanoparticles were created by femtosecond laser ablation of a gold target in flowing deionized water. This schematic diagram was redesigned with permission from reference of Qian *et al*.[2](#_ENREF_2)

**Supplementary Note 2: Synthesis of PEGylated and RGD Peptide-conjugated Chain-like Gold Nanoparticle clusters for CNV Targeting**

**Physical Production of Spherical Colloidal Gold Nanoparticles (GNPs)**

We first produced raw capping agent-free spherical colloidal GNPs used for the fabrication of CGNP clusters in the present study via a physical method of femtosecond laser ablation of a gold target as previously described in the literatures. This method uses tightly focused micro-joule (μJ) femtosecond laser pulses to produce nanoparticles and the size/size distribution of generated nanoparticles can be precisely controlled by optimizing laser parameters, such as wavelength, pulse fluence, duration, and repetition rate as shown in Fig. S19.

Briefly, the ytterbium-doped femtosecond fiber laser (FCPA μJewel D-1000, IMRA America, Ann Arbor, MI) operating at 1.045 μm delivered pulsed laser at a repetition rate of 100 kHz with 10 μJ pulse fluence and 700 fs pulse duration. The emitted laser beam was first focused by an objective lens and then reflected by a scanning mirror to the surface of the bulk gold target, which was submerged in flowing deionized water (18 MΩcm). The size of the laser spot on the gold target was estimated to be 50 μm and its position was precisely controlled by the scanning mirror. A translation stage was employed to produce relative movements between the laser beam and the gold sample in the ablation process. During the pulsed laser ablation, GNPs were partially oxidized by oxygen present in solution. These Au-O compounds were hydroxylated, followed by a proton transfer to give a surface of Au-O- as described by Sylvestre, J.-P. et al.[5](#_ENREF_5). Therefore, the GNPs produced using the laser ablation method are naturally negatively charged and no capping agents and stabilizing ligands are required for maintaining their colloidal stability. This unique feature of having capping-agent free surface for the GNPs produced this way compared with chemically synthesized GNPs allows versatile surface modification to obtain controllable surface chemistry[2](#_ENREF_2), which is crucial for self-assembling them into one dimensional (1D) CGNP clusters as it will be explained later on in this paper.

Colloidal GNPs with an average diameter of 20 nm were produced and used in our experiments. The generated nanoparticles have a narrow size distribution and have an absorption peak at 520 nm due to localized surface plasmon resonance (LSPR).

**Fabrication of CGNP clusters from spherical GNPs**

The self-assembly of spherical GNPs into CGNP clusters in aqueous solution was performed by modifying surface of GNPs with two different types of ligands, pentapeptide with an amino acid sequence CALNN and cysteamine, in a sequential manner by first mixing the colloidal GNPs with CALNN and then cysteamine. The binding of CALNN peptides and cysteamine to the GNPs is possible due to strong anchoring of the Au-sulfur bonds, which covalently attach two ligands to the surface of GNPs. CALNN peptides were bound onto the nanoparticles because they are well-known ligands for improving nanoparticle colloidal stability via enhancing interparticle electrostatic repulson[6](#_ENREF_6), which is very critical to achieve a right balance between the repulsive potential and attractive potential after addition of cysteamine molecules, a governing factor in linker-mediated self-assembly of NPs[6](#_ENREF_6),[7](#_ENREF_7). Cysteamine molecules, containing two reactive terminal groups, sulfhydryl (-SH) and amine (-NH2), can link or bridge GNPs via attaching to their surface by either covalent bonds (-SH groups) or electrostatic attraction (-NH2) thereby forming 1D CGNP clusters. It is worth noticing that only minimum amount of CALNN peptides (CALNN/GNP = 2000) and cysteamine molecules (cysteamine/GNP = 1800) necessary for inducing structurally stable chain formation were used for the surface modification of GNPs. In this way, enough space will be left on the surface of CGNP clusters for subsequent PEGylation and RGD peptide conjugation.

In a typical process, colloidal GNPs with an average diameter of 20 nm was mixed with a solution of CALNN peptides to achieve a defined molar ratio of 2000:1 between CALNN peptides and GNPs. The mixture of GNPs and CALNN peptides was kept undisturbed for 2 h at room temperature to enable sufficient conjugation of CALNN peptides to the GNPs via Au-sulfur bonds. Following the conjugation of CALNN peptides, surface of GNPs was further modified with cysteamine molecules by mixing with cysteamine solution to achieve a molar ratio of 1800 between cysteamine molecules to GNPs. The solution was kept undisturbed until the observation of significant color change from red-pink to blue, typically occurring at 24 h or serval days after addition of cysteamine molecules, which is a clear evidence of a successful self-assembly of GNPs into CGNP clusters. After formation of CGNP clusters, they were spun down to a pellet using a centrifuge and the final OD was adjusted to ~10 by adding DI water to the pellet after removing the supernatant.

**Functionalization of CGNP clusters with both PEG and RGD Peptides**

Following the methodology developed in our previous work[2](#_ENREF_2), the raw CGNP clusters were functionalized with both PEG molecules and RGD peptides for effective and selective targeting CNV in rabbit’s eyes. PEG molecules were used because they can improve stability, biocompatibility, and simultaneously minimize nonspecific interactions with biological tissues under physiological conditions by providing a hydrophilic steric barrier. RGD peptides were coated onto surface of CGNP clusters because they can specifically bind with complementary proteins, such as α5 and αv integrins. The functionalization was performed in a sequential manner by first mixing raw CGNP clusters with PEG 2k-SH and then RGD peptides, both of which contain reactive terminal -SH groups.

For 5 mL stable colloidal solution of CGNP clusters with OD 10 at 650 nm, 20 uL PEG 2k-SH solution with concentration of 1 milliMolar (mM) was added and mixed well. Our experiments confirmed that treatment of raw colloidal solution of CGNP clusters with this amount of PEG 2k-SH molecules keeps the them stable under physiological conditions and at the same time, avoids excessive surface coverage for leaving enough unoccupied space on the surface of the CGNP clusters for subsequent RGD peptides conjugation. The mixture of CGNP clusters and PEG 2k-SH was kept undisturbed for 2 h at room temperature to enable sufficient conjugation of PEG 2k-SH molecules to the CGNP clusters via Au-sulfur bonds. After this reaction, partially PEGylated CGNP clusters were further conjugated with RGD peptides by adding to them 60 µL RGD peptide solution with concentration of 1 mM. The resultant solutions were allowed to stand for another 2 h at room temperature to ensure sufficient conjugation of RGD peptides onto unoccupied space of the CGNP clusters. The final solution was transferred into a 15 mL centrifugal tube and centrifuged at 1000 g for 0.5 h until a pellet was formed. The final OD of the colloidal solution of PEGylated and RGD peptide-conjugated CGNP clusters was adjusted to ~100 by resuspending the pellet with 4 mM borate buffer (pH 8.2) containing 5 mg/ml BSA after removing the supernatant.

**Supplementary Note 3: Characterization of CGNP clusters-RGD:**

The synthesized colloidal CGNP clusters-RGD were characterized by an array of analytic instruments and techniques, including TEM, UV-Vis absorption spectroscopy, DLS and FTIR. TEM was used to visualize the fabricated CGNP clusters. TEM image of the colloidal CGNP clusters-RGD were recorded at an accelerating voltage of 100 kV. To select the excitation laser wavelength for PAM, the UV-Vis absorption spectrum of CGNP clusters-RGD was measured from 350 to 800 nm. DLS mea­surement was employed to measure hydrodynamic diameter of GNPs and CGNP clusters-RGD. The infrared spectra of PEG 2k-SH, bare CGNP clusters, and CGNP clusters-RGD were measured using FTIR for confirming the presence of PEG on the surface of CGNP clusters-RGD. All measurements and processes were carried out at room temperature, approximately 20°C.

**Supplementary Note 4: Cytotoxicity assessments**

**Cell culture:**

Bovine retinal endothelial cells (BRECs) and bovine brain endothelial cells (b.End3) were provided by the generous assistance of Dr. David Antonetti. HeLa cells were obtained from ATCC (ATCC, Manassas, Virginia, USA). Macrophage (RAW 264.7) cells were provided by the generous assistance of Dr. Raoul Kopelman. The BRECs, b.End3, Raw 264.7, and Hela cells were cultured as monolayers in 100-mm culture dishes. DMEM, which was supplemented with 10% FBS and antibiotics, was utilized as the culture medium for the b.End3, Raw 264.7, and HeLa cells. However, the protocol and medium for culturing of BRECS is different from b.End3, Raw 264.7, and HeLa cells. Prior to culturing BRECs, the culture plates were coated with fibronectin at a concentration of 1 µg/mL and kept for 1-4 hours at room temperature. Complete MCDB-131, which were supplemented with 10% FBS, 1.18 g sodium bicarbonate, 20 ng/mL EGF, 200 mg EndoGRO, 90 mg heparin, 1 mL tylosin, and 10 mL antibiotics/antimycotics, were prepared as the culture medium for the BREC cells. The cells were cultured at 37°C in humidified atmosphere of 5% CO2 and 95% air. For b.End3, Raw 264.7, and HeLa cells, the culture medium in the cell plates was changed 2-3 times per week. In contrast, the culture medium in the cell plates was changed every day for BRECs. When the BREC, b.End3, and HeLa cells in the culture dish reached 70-90% confluence, they were collected by adding 3 mL of 0.25% trypsin-EDTA solution into dishes and incubated for 3 min. The Raw 264.7 cells were isolated by using a cell scrapper. The harvested cells were then centrifuged at 500 rpm for 5 min.

***In vitro* biocompatibility of CGNP clusters-RGD (Cell viability):**

The cytotoxicity and biocompatible properties of CGNP clusters-RGD were assessed on BREC, b.End3, Raw 264.7, and Hela cells. These cells were cultured in 96-well microplates at an estimated density of 2$\times$104 cells/well in 100 µl of culture medium. The cells were incubated at 37 °C in a humidified atmosphere of 5% CO2 and allowed to grow until 80% confluence was reached. Then, the cells were wash with cold PBS and the culture medium was replaced with fresh medium containing CGNP clusters-RGD at various final concentrations (i.e., 12.5, 25, 50, 100, 200, 400, and 500 µg/mL). Control cells were used without using CGNP clusters-RGD. The cells were further incubated for 24 h and 48 h at 37 °C in humidified atmosphere of 5% CO2 to determine the effect of the incubation time on cell survival. The toxicity effect of PEG-GNPswas evaluated by using a standard methyl tetrazolium (MTT) assay.

**MTT assay**

After treatment of cells with CGNP clusters-RGD of various concentrations and incubation at different times, the treated cells were wash with cold PBS. Then, the cells were supplemented with 100 µl MTT reagent (1 mg/mL) in medium, covered with foil, and maintained in the dark for another 4 h. After the incubation, mitochondrial succinate dehydrogenase in live cells transformed MTT into visible purple formazan crystals upon the further incubation at 37 °C and was observed under the microscope. The formazan crystal was dissolved by 100 µL of DMSO. The 96-well plates were shaken gently and maintained at room temperature for 20 min to homogenize the color distribution prior to measuring the optical density (OD) at 570 nm by using an ELISA micro-plate reader (SpectraMax, 340, Molecular Device, Sunnyvale, CA, USA). The relative cell viability was calculated and compared with that of non-treated blank group by using the following formula:

$\text{P = }\frac{\text{OD experimental group}}{\text{OD control group}}\text{ × 100}$ (1)

**Apoptosis assay**

**Microscope analysis: Hoechst, PI, Annexin-V FITC triple staining**

To assess the changes of cell such as cell morphology, apoptosis, necrosis, and cell population, fluorescence microscopy analysis was performed by using Hoechst 33342, propidium iodide (PI), and Annexin-V FITC triple staining. The cells with homogeneously stained nuclei were considered viable, and the cells with chromatin condensation or fragmentation were considered apoptotic. The Hoechst 33342 stains the nuclei of healthy cells, the apoptotic cells, as well as the necrotic cells and emits blue fluorescence light. The Annexin-V FITC typically identifies early apoptotic cells in a population of cells via binding to cell surface expressing phosphatidylserine, an early apoptosis maker. Cells stained with Annexin V-FITC are visualized as a bright green color by a fluorescence microscope. In contrast, late apoptotic or necrotic cells are differentiated with using PI, which only stains DNA and RNA inside of dead cells or the ones with reversibly damaged membranes and generates rad fluorescence. Hoechst 33342, PI, and Annexin-V FITC triple staining was performed on BREC, b.End3, and HeLa cells. A density of 4$\times$105 cells/well was seeded in 33 mm µ-dish culture plates and incubated for 24 h at 37 °C in a humidified atmosphere of 5% CO2. After 24 h incubation, the medium in the plates was discarded, and the cells were then washed with cold PBS and treated with fresh media containing CGNP clusters-RGD at final concentration of 200 µg/ml and further incubated for 4 h. A control cell sample was prepared without incubation with nanoparticles. After the incubation, the treated cells were washed three times with PBS to remove free unbound nanoparticles before triple staining with Hoechst 33342, PI, and Annexin-V FITC fluorescent dye. After washing, 500 µl 1X binding buffer was added to the cells. Then, the cells were stained by an Annexin-V FITC Apoptosis Detection Kit (BD Biosciences, CA, USA) including a mixed solution of 5 µl of Annexin-V FITC and 5 µl of PI. The cells were covered with foil and incubated for another 15 min in the dark environment. The stained cells were washed two times with PBS and then fixed with 4% formaldehyde and incubated for 20 min at 37 °C. Next, the cells were washed with cold PBS two times, and then, 300 µlof 10 µg/ml Hoechst 33342 solution was added to the cells and incubated for another 20 min at 37 °C in the dark environment. Finally, the stained cells were washed three times with cold PBS and examined under a Leica SP5 laser scanning confocal fluorescence microscope (Leica SP5, Wetzlar, Germany).

**Quantitative analysis: Flow cytometric analysis**

To evaluate the degrees of apoptosis and necrosis after treating cells with CGNP clusters-RGD, flow cytometry was implemented using an Annexin-V FITC Apoptosis Detection Kit. The flow cytometric analysis is typically used to determine the change in granularity and size of the cells. Different from necrosis, apoptosis usually involves the changes of the cell morphology such as nuclear fragmentation, cell shrinkage, and DNA loss. Necrotic and apoptotic cells can be classified using the light scattering effect of the cells. For flow cytometric analysis, after cells treated with CGNP clusters-RGD being incubated for 24 and 48 h, they were trypsinized and harvested using centrifugation at 1500 rpm for 3 min. Then, the cells were re-suspended in 500 µl of 1X binding buffer. 10 µg/ml of Annexin-V FITC and PI solution were added to the cell suspension and incubated for exactly 15 min at room temperature in the dark environment. Then, the cells were diluted with 1 ml of cold PBS and transferred to 1.5 ml glass tube for flow cytometer. In addition, three staining samples including untreated cells, cells stained with Annexin-V FITC and cells stained with PI were used as control. All cells samples were immediately measured with a flow cytometer. According to the apoptosis protocol, the analysis provided quantitative information on the cellular status in four quadrants: Lower left portion (Q1) denotes viable cells ($\text{Annexin-V}^{\text{-}}\text{/PI}^{\text{-}}$); lower right (Q2) is early apoptotic cells ($\text{Annexin-V}^{\text{+}}\text{/PI}^{\text{-}}$), upper right (Q3) represents late apoptotic cells ($\text{Annexin-V}^{\text{+}}\text{/PI}^{\text{+}}$), and upper left (Q4) indicates necrotic cells ($\text{Annexin-V}^{-}\text{/PI}^{+}$).

**Cellular uptake**

The cellular uptake of the CGNP clusters-RGD was performed on the BRECs, b.End3, Raw 264.7, and HeLa cells. In order to evaluate the cellular uptake of the CGNP clusters-RGD, the cells were cultured in a 35 mm microplates at an estimated density of 2$\times$105 cells/mL in a 1 mL of culture medium and incubated for 24h. After 24h incubation, the cells were wash with cold PBS and the culture medium was replaced with fresh medium containing CGNP clusters-RGD coated with FITC at a final concentration of 50 µg/mL and incubated for another 24 h. After the incubation time, the cultured cells were washed three times with PBS to remove free unbound nanoparticles before staining with Hoechst 33342 fluorescent dyes. After washing, the cells were fixed with 4% formaldehyde and incubated for 20 min at 37 °C. Next, the cells were washed with cold PBS two times, and then, 500 µLof 10 µg/mL Hoechst solution was added to the cells and incubated for another 20 min at 37 °C in the dark environment. Finally, the stained cells were washed three times with cold PBS and the cell morphology was captured with a Leica SP5 laser scanning confocal fluorescence microscope (Leica SP5, Wetzlar, Germany).

For quantitative analysis of the intracellular uptake of CGNP clusters-RGD, after cells treated with CGNP clusters-RGD at concentration of 50 µg/ml being incubated for 24, 48, and 72 h, they were trypsinized and harvested using centrifugation. The collected cells were dissolved at elevated temperature (100 oC) in a 1 ml concentrated nitric acid (67%) and 3 ml hydrochloric (37%). The amount of the nanoparticles cells uptake was determined using SQ-ICP-MS (iCAP™ RQ ICP-MS, Thermo Fisher Scientific, Bremen, Germany).

**Stability of CGNP clusters-RGD**

**Stability of CGNP clusters-RGD in vitro at the cellular level (Dark Field images)**

Stability assessment of CGNP clusters-RGD in terms of their structure and morphological integrity in vitro at the cellular level was examined using HeLa cells. HeLa cells were treated with conventional colloidal spherical GNPs with diameter of 20 nm and with CGNP clusters-RGD at the same concentration of 50 µg/ml and incubated for 24 h. The treated cells were washed with cold PBS to remove unattached nanoparticles and fixed with 4% formaldehyde and incubated for 20 min at 37 °C. Dark field images were captured with an Olympus GX71 inverted microscope in order to confirm the structure and morphological integrity of CGNP clusters-RGD in vitro at the cellular level.

**Photostability of CGNP clusters-RGD**

To evaluate the photostability of CGNP clusters-RGD under laser irradiation, a group of four samples was prepared. Each sample contained 100 µl CGNP clusters-RGD at concentration of 5 mg/ml. These samples were illuminated with nanosecond pulsed laser at different pulse fluences (0.005, 0.01, 0.02, and 0.04 mJ/cm2) at the wavelength of 650 nm. After this illumination, their UV-Vis absorption spectra were measured.

**Circulation time of CGNP clusters-RGD**

The circulation kinetics of the synthesized CGNP clusters-RGD in blood was performed in rabbits *in vivo*. The rabbits were intravenously injected with CGNP clusters-RGD at two different concentrations (2 mg/ml and 4 mg/mL) via ear vein. To evaluate the circulation kinetics, 2 mL of blood were collected using 26-gauge needle and 1 mL syringe. The blood samples were collected before and after injection of CGNP clusters-RGD at 15, 30, 45, 60 min, 2, 4, 8, 12, 24, 48, and 72 h. The collected blood samples were kept in 5 ml heparin coated glass tube. Then, the collected blood samples were dissolved in concentrated nitric acid and hydrochloric acid for analyses by inductively coupled plasma mass spectrometer (ICP-MS). Analyses of diluted blood was performed on a SQ-ICP-MS ((iCAP™ RQ ICP-MS, Thermo Fisher Scientific, Bremen, Germany).

***In Vivo* Toxicity**

We investigate the toxicity of CGNP clusters-RGD in laboratory mice. For this investigation, nine groups of the animals were prepared including control group (N=3), groups injected with bare colloidal GNPs, and CGNP clusters-RGD at concentrations of 2.5, 5, 10, and 20 mg/kg (N=24). 500 µL of each freshly prepared solution were injected via the tail vein. Then, the mouse was returned to the animal facility and the body weight was monitored every day for 7 days. At day 7 after injection, all the animals were euthanized. Blood and organs such as heart, lung, kidney, liver, and spleen were collected. The organs were briefly washed with PBS, weighted accurately, and were stored in a freezer at temperature of -40 oC for determination the amount of gold by ICP-MS. Blood samples were kept in 600 µl blood collection tubes. Then, all the samples were centrifuged at 1300 rpm for 10 min. After centrifuged, the samples were stored in a refrigerator at 4 oC for mini chemistry panel analysis.

**Biodistribution: ICP-MS Method**

A west digestion protocol was used to process and digest the tissues sample for ICP-MS quantification as described in detailed in references 1, 10 and 11with modifications. Briefly, 2 mL of the fluid sample or 0.2 g of organ sample was dissolved at elevated temperature (100 oC) in 10 mL concentrated 67% nitric acid for 30 minutes. Subsequently, 30 mL hydrochloric acid was added and then heated to dryness for 3 more hours until the mixed solution reached 1 mL. The melted solutions solution was diluted with 4 mL DI water and filtered with 0.22 µL cellulose nitrate syringe filter (Merc Millipore Ltd., Co. Cork, Ireland) and dissolved in 5 mL deionized water. All obtained samples were then analyzed on inductively coupled plasma mass spectrometer (ICP-MS) for gold content using a standard calibration. A blank and three standards were used for calibration. Analyses of diluted blood and tissue samples were performed on a SQ-ICP-MS ((iCAP™ RQ ICP-MS, Thermo Fisher Scientific, Bremen, Germany).

**Histological analysis**

To quantify the toxicity of CGNP clusters-RGD for *in vivo* PAM and OCT and to evaluate the change in retinal vessels, rabbits were euthanized fourteen days after intravenous infusion of CGNP clusters-RGD. The eyes and other vital organs were extracted from the euthanized rabbit for histological analysis. The eye and different tissues from the treated group and control group were removed aseptically from the euthanized rabbits. The isolated samples were fixed in 10% neutral buffered formalin (VWR, Radnor, PA) for a minimum of 48 h. To prevent retinal detachment, the isolated eyeball was fixed in Davidson’s fixative solution (Electron Microscope Sciences, PA, USA) for 24 hours. The sample was transferred to 50 % of alcohol solution (Fisher Scientific, PA, USA) for an additional 8 hours. Finally, the sample was changed to 70 % alcohol solution and kept at room temperature for 24h prior to embedding in paraffin. The fixed tissues were cross-sectionally cut in 5 mm sections and embedded in paraffin. Subsequently, the paraffin-embedded tissues were sliced to a thickness of 4 µm and stained with hematoxylin and eosin (H&E) and terminal deoxynucleotidyl transferase dUTP nick end labeling (TUNEL) assay using a Leica autostainer XL (Leica Biosystems, Nussloch, Germany) under standard conditions. The slides were examined by using a Leica DM600 light microscope (Leica Biosystems, Nussloch, Germany) to detect apoptotic or necrotic cells.

**Electron Microscopy**

For electron microscopy, liver tissues were collected and divided into pieces of approximately 0.5 x 0.5 x 2 mm3 using a razorblade. Then, the samples were fixed in 2.5% glutaraldehyde in a 0.1 M Sorensen’s phosphate buffer (pH = 7.4) and stored in a refrigerator at 4 oC for a minimum of 24 h. Then, the samples were rinsed three time for 15 min each with 0.1 M Soren’s buffer and postfixed for 1 h at room temperature in 1% osmium tetroxide in 0.1 M Sorensen’s buffer. After post-fixation, the samples were rinsed three times with 0.1 M Sorensen’s phosphate buffer for 15 min. The samples were dehydrated in 25, 50, 70, 95 and 100% of acetone for 5 min. Then, the samples were polymerized at 60 oC for 24h. The processed samples were cut into ultrathin (~70 nm) slices using ultra-microtome. Ultrathin sections were mounted onto 200 mesh fine bar hex grids without the base layers and were stained with uranyl acetate and lead citrate at a temperature of 56 oC for 10 min. The specimens were examined, and electron micrographs were taken with transmission electron microscope (JOEL-JEM 1400 Plus, Japan Electron Optic, Tokyo, Japan)

**Supplementary Note 5: Sample Preparations**

**Phantom preparation:**

A group of phantom samples was prepared and used to examine PAM and OCT response of synthesized CGNP clusters-RGD. For PAM, the phantom was made of silicone tube with an inner diameter of 0.30 mm and outer diameter of 0.64 mm (N = 6). The tubes were filled with different concentration of CGNP clusters-RGD solution (i.e., 0 (saline), 0.005, 0.01, 0.02, 0.04, and 0.08 mg/mL) by a 30-gauge 1mL insulin syringe (Covidien, MA, USA). To compare the PA signal between blood and CGNP clusters-RGD, blood was mixed with CGNP clusters-RGD at final concentration of 0.02 and 2.5 mg/mL. The blood and mixture of blood and CGNP clusters-RGD were injected into silicone tubes. Both the distal ends of each tube were sealed with optical adhesive. Prior to PAM, the phantoms were placed on the top of the coverslip. The coverslip was then placed in a degassed water tank to prevent any cavitation. For OCT, CGNP clusters-RGD suspension solution was filled into capillaries glass tubes (inner diameter = 0.30 mm and outer diameter = 0.54 mm) at various concentrations (i.e., 0 (saline), 0.005, 0.01, 0.02, 0.04, and 0.08 mg/mL) (N = 6). In order to evaluate the OCT signal enhancement between blood and CGNP clusters-RGD, CGNP clusters-RGD was diluted with blood at final concentration of 0.02 and 0.04 mg/mL. Both the distal ends of each tube were capped with optical adhesive.

**Animal model preparation:**

All rabbit studies were employed under the guidelines of the ARVO (The Association for Research in Vision and Ophthalmology) Statement on the care and use of laboratory animals in Ophthalmic and Vision Research. The experimental protocol was approved by the Institutional Animal Care and Use Committee (IACUC) of the University of Michigan (Protocol number: PRO00008566, PI: Y. Paulus). New Zealand White rabbits that were 2-3 months old and weighed 1.8-2.8 kg were bred from the North Campus Laboratory Animal Science at the University of Michigan and used for the experiments.

**Supplementary Note 6: Retinal disease models:**

**Retinal vein occlusion (RVO) model:**

The retinal vein occlusion model was performed as described previously by Oncel *et al*.[12](#_ENREF_12) and Nguyen *et al.*[*13*](#_ENREF_13). In brief, a contact lens (Volk H-R Wide Field, laser spot 2x magnification, Volk Optical Inc, Mentor, OH, USA) was placed on the cornea of the rabbit eye with Gonak Hypromellose Ophthalmic Demulcent Solution 2.5% (Akorn, Lake Forest, IL, USA) used for coupling. Rose Bengal was intravenously injected into the rabbit via the marginal ear vein. 5 seconds after the Rose Bengal injection, the rabbit eye was illuminated with a laser beam at the wavelength of 532 nm and power of 150-300 mW. (Vitra 532nm, Quantel Medical, Cournon d’Auvergne, France). The laser beam size was approximately 75 µm in aerial diameter and the irradiation time was 0.5 s per spot. Laser irradiation occurred at a distance of a half to one disc diameter from the optic disc margin. Twenty shots of the laser were applied at the same position on the retinal vein at the laser power of 150 mW until the blood flow was completely stopped and observed in the vein. Then, the laser power was increased to 300 mW and applied for further 20 shots to prevent reperfusion of the vein[14](#_ENREF_14).

**Subretinal injection of vascular endothelial growth factor (VEGF) model:**

To create the model of subretinal injection of VEGF, a mixture of 750 ng Human VEGF-165 (100 µg/mL) (Shenandoah Biotechnology, Warwick, USA) in 20 µL of Matrigel (Corning, NY, USA) were used to inject into the rabbit eyes.

**Supplementary Note 7: Animal model monitoring**

**Color fundus photography**

The major retinal vessels before and after laser irradiation and subretinal injection were imaged using a 50-degree color fundus photography (Topcon 50EX, Topcon Corporation, Tokyo, Japan). The color fundus images were used to select the target vessels for laser irradiation, to monitor the position of photothrombotic after treatment, and to select the position for subretinal injection. The dynamic changes of retinal blood vessels and the percentage of blood perfusion before and after laser illumination as well as the vessel density at the subretinal injection areas were obtained from the color fundus images.

**Fluorescein angiography (FA)**

Following color fundus images, fluorescein sodium at concentration of 10% fluorescein (Akorn, Lake SegForest, IL, USA) was administrated intravenously in the rabbit marginal ear vein at a dose of 0.2 mL. The FA images were subsequently obtained immediately during the transit phase after injection. Late phase fluorescence photos were acquired at least every minute for a period of at least 15 minutes. Fluorescein angiography (FA) was utilized to evaluate the vasculature and to confirm vascular occlusion.

**Multimodal photoacoustic microscopy (PAM) and optical coherence tomography (OCT) imaging system:**

A custom-built dual modality PAM and OCT system (Figure S1) was used for the imaging studies. PAM utilizes an optical parametric oscillator (OPO) pumped by a diode-pumped Q-switched Nd:YAG laser (NT-242, Ekspla, Lithuania, pulse repetition rate 1 kHz, duration 3 – 6 ns, tunable wavelength range 405 – 2600 nm). The laser light from OPO was perpendicularly reflected at the prism and then spread through a through a half-wave plate attenuator mounted on a motorized rotation stage; it was then focused, filtered, and collimated by a beam collimator. The beam collimator is composed of a focusing lens (focal length 250 mm), a pinhole (diameter 50 μm), and a collimating lens (focal length 30 mm). After collimation, the circular-shaped pattern was formed with a diameter of approximately 2 mm. The circular-shaped light was then split with a ratio of 90/10 (reflection/transmission). The transmitted portion was recorded by a photodiode for pulse-to-pulse laser fluence monitoring. The reflected portion was successively deflected by a mirror and a dichroic mirror (DM) and raster-scanned by a two-dimensional galvanometer, which is a shared component with the spectral domain (SD)-OCT system. The scanned beam traveled through a telescope consisting of a scan lens (focal length 36 mm) and an ophthalmic lens (OL, focal length 10 mm) and was finally focused on the fundus by the rabbit eye optics. The laser light fluence on the eye used to acquire images was 0.01 mJ/cm2 at 578nm, which is half of the American National Standards Institute limit[15](#_ENREF_15). To detect the laser-induced acoustic signals, a custom-made needle-shaped ultrasound transducer with a central frequency of 27.0 MHz (Optosonic Inc., Arcadia, CA, USA) was mounted in contact with the conjunctiva of the central visual axis and aligned to enable accurate alignment with laser light. The axial and transverse resolutions were 37.0 and 4.1 µm, respectively. The received analog photoacoustic signals were filtered and amplified using a low-noise amplifier (AU-1647, L3 Narda-MITEQ, NY). After the amplification, the signals were converted into digital signals and recorded using a high-speed digitizer at a sampling rate of 200 MS/s (PX1500-4, Signatec inc., Newport Beach, CA). The recorded data was then used to reconstruct two-dimensional (2D) or three-dimensional (3D) images of the eye blood vessels. 2D depth-sensitive PAM images were acquired by implementing horizontal scanning lines along the x-axis. For 2D image reconstruction, each sample was scanned along x- and y-directions using an optical-scanning galvanometer with a resolution of 2.5 × 5 µm2, while scanning depth (z-direction) was fixed at the focal depth of the imaging transducer. For a 1.5 × 1 mm2 field of view, the acquisition time was approximately 60 s. In addition, by performing faster scanning along the y-axis, the volumetric PA images were obtained accordingly. To assess the potential of the fabricated nanoparticles for the enhanced PA image contrast, the image contrast, which was defined as the difference between the PA signal of the targeted area and its background, was estimated from the reconstructed images as described in previous studies[17-19](#_ENREF_17). Additionally, to visualize the margin of the blood vessel, 3D image reconstruction was performed as a post image processing analysis. The 3D image reconstruction was performed on a set of 256 A-scan images with a gap of 2.5 µm between the consecutive slides. These images were selected, aligned, and combined using Amira software. Further post-processing was performed on the 3D image to improve visualization of the newly formed blood vessels margin.

Optical Coherence Tomography (OCT) was performed from a commercially available OCT system (Ganymede-II-HR, Thorlabs, Newton, NJ) by adding the ocular lens after the scan lens and a dispersion compensation glass in the reference arm. A combination of two super luminescent diodes with center wavelengths of 846 nm and 932nm was used to excite the tissue. The lateral and axial resolutions are 4 µm and 3 µm, respectively. The OCT light source was coaxially aligned with the PAM system. Thus, OCT can be used as guided PAM and help interpret PAM results. Fundus photography was performed using the Topcon TRC 50EX fundus camera (Topcon Corporation, Tokyo, Japan).

***In vivo* PAM/OCT for retinal and choroidal neovascularization evaluation:**

Multimodal PAM and OCT were used to evaluate the retinal and CNV pre and post intravenous (I.V.) injection of the synthesized CGNP clusters-RGD. All PAM and OCT analyses of the retinal and neovascularization with the integration of nanoparticles for comparison of signal enhancement pre- and post-administration were conducted as blinded and randomized experiments. Twelve New Zealand White rabbits (~2.5 kg) were used for all the imaging studies to detect the margin of CNV. Prior to the experiments, animal state, mucous membrane color, temperature, heart rate, and respiratory rate were monitored and recorded as a general procedure by using a pulse oximeter (Smiths Medical, MN, USA). Then, a mixed solution of ketamine (40 mg/kg IM, 100 mg/mL) and xylazine (5 mg/kg IM, 100 mg/mL) was used to fully anesthetize each rabbit model with an intramuscular application. To sustain anesthesia during the *in vivo* experiments, a vaporized isoflurane anesthetic (Surgivet, MN, USA) was applied to provide 1 L/min oxygen and 0.75% isoflurane. The pupils of rabbit were diluted using tropicamide 1% ophthalmic and phenylephrine hydrochloride 2.5% ophthalmic. Topical tetracaine 0.5% was instilled in the eye for topical anesthesia in addition to lubricant (Systane, Alcon Inc., TX, USA) to prevent dehydration of the cornea. The PAM and OCT images of CNV were obtained before and after the I.V injection of GNP (0.4 mL, 5 mg/mL). A water-circulating blanket (TP-700, Stryker Corporation, Kalamazoo, MI) was used to maintain the temperature of the rabbit during the experiment. After the anesthetic injection, the rabbits were positioned on the imaging platform, and the areas of interest were monitored by the fundus camera. The head and body of the rabbit were placed on different high performance, custom-made stabilization platforms to minimize breathing and other motion artifacts. The rabbit vessels were first imaged with the OCT system. Then, an ultrasound transducer was mounted in the eye chamber, allowing it to move freely in 3D while not applying any physical pressure on the rabbit eyes. The ultrasound gel was sandwiched between the eye and the transducer. Then, the targeted regions were selected by the fundus camera and imaged with PAM. After acquiring the control PAM image, CGPs-cluster RGD (0.4 mL, 5 mg/mL) were intravenously injected into the rabbit with a 1 mL syringe, 27-gauge needle in the marginal ear vein. PAM, OCT, FA, color fundus photograph images of the injected rabbit were performed at different time points after injecting nanoparticles. After the *in vivo* experiments, vitals including mucous membrane color, heart rate, respiratory rate, and rectal temperature were monitored and recorded until the rabbit fully recovered. Then, the rabbit was immediately returned to its rack and monitored the body weight every day for 7 days. The three-dimensional structure of blood vessels was also reconstructed to visualize the structure and to estimate the detected diameter of blood vessels by using Amira software.

**Supplementary References:**

1 Mankovskii, G. & Pejović-Milić, A. Comparison of total reflection X-ray fluorescence spectroscopy and inductively coupled plasma for the quantification of gold nanoparticle uptake. *Spectrochimica Acta Part B: Atomic Spectroscopy*, 105764 (2020).

2 Qian, W., Murakami, M., Ichikawa, Y. & Che, Y. Highly efficient and controllable PEGylation of gold nanoparticles prepared by femtosecond laser ablation in water. *The Journal of Physical Chemistry C* **115**, 23293-23298 (2011).

3 Liu, B., Hu, Z., Murakami, M. & Che, Y. (Google Patents, 2012).

4 Liu, B., Hu, Z., Che, Y., Chen, Y. & Pan, X. Nanoparticle generation in ultrafast pulsed laser ablation of nickel. *Applied Physics Letters* **90**, 044103 (2007).

5 Sylvestre, J.-P. *et al.* Surface chemistry of gold nanoparticles produced by laser ablation in aqueous media. *The Journal of Physical Chemistry B* **108**, 16864-16869 (2004).

6 Lévy, R. *et al.* Rational and combinatorial design of peptide capping ligands for gold nanoparticles. *Journal of the American Chemical Society* **126**, 10076-10084 (2004).

7 Nel, A. E. *et al.* Understanding biophysicochemical interactions at the nano–bio interface. *Nature materials* **8**, 543 (2009).

8 Lizard, G. *et al.* Kinetics of plasma membrane and mitochondrial alterations in cells undergoing apoptosis. *Cytometry* **21**, 275-283 (1995).

9 Gschwind, M. & Huber, G. Apoptotic Cell Death Induced by β‐Amyloid1–42 Peptide Is Cell Type Dependent. *J. Neurochem.* **65**, 292-300 (1995).

10 Takeuchi, I., Nobata, S., Oiri, N., Tomoda, K. & Makino, K. Biodistribution and excretion of colloidal gold nanoparticles after intravenous injection: Effects of particle size. *Bio-Medical Materials and Engineering* **28**, 315-323 (2017).

11 Simpson, C. A., Salleng, K. J., Cliffel, D. E. & Feldheim, D. L. In vivo toxicity, biodistribution, and clearance of glutathione-coated gold nanoparticles. *Nanomedicine: Nanotechnology, Biology and Medicine* **9**, 257-263 (2013).

12 Oncel, M., Peyman, G. A. & Khoobehi, B. Tissue plasminogen activator in the treatment of experimental retinal vein occlusion. *Retina (Philadelphia, Pa.)* **9**, 1-7 (1989).

13 Nguyen, V. P., Li, Y., Zhang, W., Wang, X. & Paulus, Y. M. Multi-wavelength, en-face photoacoustic microscopy and optical coherence tomography imaging for early and selective detection of laser induced retinal vein occlusion. *Biomed Opt Expess* **9**, 5915-5938 (2018).

14 Ameri, H., Ratanapakorn, T., Rao, N. A., Chader, G. J. & Humayun, M. S. Natural course of experimental retinal vein occlusion in rabbit; arterial occlusion following venous photothrombosis. *Ger J Ophthalmol* **246**, 1429 (2008).

15 Tian, C., Zhang, W., Mordovanakis, A., Wang, X. & Paulus, Y. M. Noninvasive chorioretinal imaging in living rabbits using integrated photoacoustic microscopy and optical coherence tomography. *Optics express* **25**, 15947-15955 (2017).

16 Chao Tian, W. Z., Van Phuc Nguyen, Xueding Wang, and Yannis M. Paulus. Novel Photoacoustic Microscopy and Optical Coherence Tomography Dual-modality Chorioretinal Imaging in Living Rabbit Eyes. *The Journal of Visualized Experiments* In Press (2017).

17 Nguyen, V. P. *et al.* Biocompatible astaxanthin as a novel marine-oriented agent for dual chemo-photothermal therapy. *PloS one* **12**, e0174687 (2017).

18 Luke, G. P., Yeager, D. & Emelianov, S. Y. Biomedical applications of photoacoustic imaging with exogenous contrast agents. *Ann Biomed Eng* **40**, 422-437 (2012).

19 Nguyen, V. P., Oh, J., Park, S. & Wook Kang, H. Feasibility of photoacoustic evaluations on dual‐thermal treatment of ex vivo bladder tumors. *J Biophotonics* (2016).
