# Supplementary material for: Chain-like gold nanoparticle clusters for multimodal photoacoustic microscopy and optical coherence tomography enhanced molecular imaging
Source: Nat Commun. 2021 Jan 4;12:34. doi: 10.1038/s41467-020-20276-z (PMC7782787; doi:10.1038/s41467-020-20276-z)
Supplement: Supplementary file 2 — Description of Additional Supplementary Files [file 41467_2020_20276_MOESM2_ESM.docx]

**Description of Additional Supplementary Files**

**Supplementary Movie 1:**

3D image reconstruction of retinal and choroidal neovascularization at day 3 after administration of CGNP clusters-RGD at final concentration of 0.02 mg/mL in rabbits
